# The Transcription Factor BcLTF1 Regulates Virulence and Light Responses in the Necrotrophic Plant Pathogen *Botrytis cinerea*

**DOI:** 10.1371/journal.pgen.1004040

**Published:** 2014-01-09

**Authors:** Julia Schumacher, Adeline Simon, Kim Christopher Cohrs, Muriel Viaud, Paul Tudzynski

**Affiliations:** 1 IBBP, WWU Münster, Münster, Germany; 2 INRA, BIOGER, Grignon, France; Dartmouth Medical School, United States of America

## Abstract

*Botrytis cinerea* is the causal agent of gray mold diseases in a range of dicotyledonous plant species. The fungus can reproduce asexually by forming macroconidia for dispersal and sclerotia for survival; the latter also participate in sexual reproduction by bearing the apothecia after fertilization by microconidia. Light induces the differentiation of conidia and apothecia, while sclerotia are exclusively formed in the absence of light. The relevance of light for virulence of the fungus is not obvious, but infections are observed under natural illumination as well as in constant darkness. By a random mutagenesis approach, we identified a novel virulence-related gene encoding a GATA transcription factor (BcLTF1 for light-responsive TF1) with characterized homologues in *Aspergillus nidulans* (NsdD) and *Neurospora crassa* (SUB-1). By deletion and over-expression of *bcltf1*, we confirmed the predicted role of the transcription factor in virulence, and discovered furthermore its functions in regulation of light-dependent differentiation, the equilibrium between production and scavenging of reactive oxygen species (ROS), and secondary metabolism. Microarray analyses revealed 293 light-responsive genes, and that the expression levels of the majority of these genes (66%) are modulated by BcLTF1. In addition, the deletion of *bcltf1* affects the expression of 1,539 genes irrespective of the light conditions, including the overexpression of known and so far uncharacterized secondary metabolism-related genes. Increased expression of genes encoding alternative respiration enzymes, such as the alternative oxidase (AOX), suggest a mitochondrial dysfunction in the absence of *bcltf1*. The hypersensitivity of Δ*bctlf1* mutants to exogenously applied oxidative stress - even in the absence of light - and the restoration of virulence and growth rates in continuous light by antioxidants, indicate that BcLTF1 is required to cope with oxidative stress that is caused either by exposure to light or arising during host infection.

## Author Summary

Both fungal pathogens and their host plants respond to light, which represents an important environmental cue. Unlike plants using light for energy generation, filamentous fungi use light, or its absence, as a general signal for orientation (night/day, underground/on the surface). Therefore, dependent on the ecological niche of the fungus, light may control the development of reproductive structures (photomorphogenesis), the dispersal of propagules (phototropism of reproductive structures) and the circadian rhythm. As in other organisms, fungi have to protect themselves against the detrimental effects of light, i.e. the damage to macromolecules by emerging singlet oxygen. Adaptive responses are the accumulation of pigments, especially in the reproductive and survival structures such as spores, sclerotia and fruiting bodies. Light is sensed by fungal photoreceptors leading to quick responses on the transcriptional level, and is furthermore considered to result in the accumulation of reactive oxygen species (ROS). In this study, we provide evidence that an unbalanced ROS homoeostasis (generation outweighs detoxification) caused by the deletion of the light-responsive transcription factor BcLTF1 impairs the ability of the necrotrophic pathogen *Botrytis cinerea* to grow in the presence of additional oxidative stress arising during illumination or during infection of the host.

## Introduction

*Botrytis cinerea* Persoon: Fries (teleomorph *Botryotinia fuckeliana* (de Bary) Whetzel) is a necrotrophic pathogen with a broad host range causing gray mold disease in several economically important plants including grape vine and strawberry [1]–[2]. Sources of infection by *B. cinerea* are the conidia that are ubiquitously distributed in the air. After landing on the plant surface, the conidia germinate and form short germ tubes which directly penetrate. Infection may also occur from already established mycelia, in this particular case, multicellular infection structures (infection cushions) are formed. Usually, after penetration the epidermal and underlying cells die and *B. cinerea* establishes a primary (restricted) infection. Then, the fungus starts a massive outgrowth (spreading) that results finally in the maceration of the plant tissue (soft rot) and formation of conidia for new infections. During the interaction, the fungus produces phytotoxic metabolites e.g. botrydial (BOT) and botcinic acid (BOA), necrosis-inducing proteins, reactive oxygen species (ROS), cell wall-degrading enzymes, peptidases and phytohormones [1], [3]–[5]. Gene replacement approaches have identified only a few virulence factors to date, probably because of their redundancy [6], [2]. Hence, the importance of the toxic secondary metabolites BOT and BOA for infection becomes apparent only when strains are lacking both toxins [7]–[9].

The asexual macroconidia formed by branched conidiophores are undisputedly the main source of primary inoculum under given environmental conditions, such as high humidity and moderate temperatures. The dark pigmented sclerotia, however, serve as survival structures e.g. for over-wintering. They may germinate vegetatively to yield new mycelia and conidia, or they can act as the female parent in sexual reproduction when microconidia (male parent) carrying the opposite mating type are present. Apothecia, containing the linear asci with the ascospores, develop on the fertilized sclerotia [10]. It is difficult to estimate how frequently sexual reproduction happens in nature; nevertheless, it is assumed that it significantly contributes to genetic variation of *B. cinerea* [11]. If isolates produce sclerotia and microconidia they can function as female and male partners in reciprocal crosses [12]; however, not all *B. cinerea* isolates form sclerotia. As early as 1929, a comparative morphological study reported on three groups of *B. cinerea* isolates predominantly forming sclerotia, sterile mycelia or conidia [13]. When isolates are able to produce both conidia and sclerotia such as the sequenced strain B05.10 [14], [2], it happens in a light-dependent fashion: full-spectrum/white light induces the formation of conidia while its absence results in formation of sclerotia, and even limited exposures to light are sufficient to suppress sclerotial development [15]. *B. cinerea* was found to respond to different wavelengths of light: UV light results in conidiation, red light promotes sclerotial development and blue light leads to the accumulation of undifferentiated aerial mycelia [15]–[16]. Based on these observations it was hypothesized in 1975 that two receptors sensing UV/blue light and red/far-red light, respectively, are involved in regulation of asexual reproduction in *B. cinerea* [17]. In accordance with this model, the inspection of the genome sequence has revealed the existence of two UV light-sensing cryptochromes, two blue light sensors, two opsin-like proteins that may sense green light as well as three red/far-red light-sensing phytochromes. Notably, the number of phytochromes has been expanded in *B. cinerea* in comparison to *Aspergillus nidulans* and *Neurospora crassa* with one and two representatives, respectively, suggesting a special role of red light in its life cycle [18], [2].

The best studied fungus in terms of light signaling is *N. crassa* that mainly responds to blue light in order to regulate mycelial carotenoid biosynthesis, conidiation, the circadian clock, and formation of protoperithecia [19]. Most of these responses are mediated via the *WHITE COLLAR* transcription factors WC-1 and WC-2 that form a complex (WCC) in response to blue light leading to the activation of gene expression [20]. In accordance with the fact that blue light is the most active wavelength in *N. crassa*, deletions of the cryptochromes, opsins and phytochromes did not result in obvious phenotypes [21]–[24]. In contrast, and similarly to *B. cinerea*, *A. nidulans* does respond to UV, blue and red light and forms asexual spores in the light, but preferentially undergoes sexual reproduction (cleistothecia) in the dark [25]–[26]. It was demonstrated that the single cryptochrome CryA represses the formation of cleistothecia in the light [27], and that blue light sensed via the WCC complex (LreA/LreB) and red light sensed via the single phytochrome FphA act in concert to trigger the formation of conidia in the light [28]. However, unlike *A. nidulans*, *B. cinerea* possesses homologues of VVD-1 and FRQ-1 that are involved in photoadaptation and the circadian clock in *N. crassa* [18], [20], [29].

In *B. cinerea*, the photoreceptors, with the exception of the opsin BOP1 whose deletion affected neither affect light-dependent differentiation nor virulence [30], have not been functionally studied so far. However, *“blind” B. cinerea* mutants will be helpful to study the role of light in the fungus-host interaction without interfering with the host's metabolism. Recently, the reason for the *“blind: always conidia”* phenotype of two natural isolates (T4, 1750) has been identified: different single nucleotide polymorphisms (SNPs) in *bcvel1* encoding a VELVET family protein result in truncated proteins and consequently in the deregulation of the light-dependent differentiation and in severely reduced virulence suggesting an interrelationship between light perception and virulence in *B. cinerea* [31]–[32].

This study describes the identification of a novel virulence-associated gene by a random mutagenesis approach using *Agrobacterium tumefaciens*-mediated transformation (ATMT). The identified gene *bcltf1* encodes a GATA-type transcription factor homologous to *A. nidulans* and *A. fumigatus* NsdD, *N. crassa* SUB-1, and *Sordaria macrospora* PRO44 for whom the involvement in differentiation programs were demonstrated [33]–[38]. To our knowledge, the functions of this GATA transcription factor are described for the first time in a plant pathogen and reveal some overlapping features with the characterized homologues with regard to regulation of reproduction. Additionally, we report on specific functions of this transcription factor with regard to maintenance of ROS homoeostasis (production vs. scavenging of ROS), secondary metabolism and virulence.

## Results

### Identification of the GATA-type transcription factor BcLTF1

**Figure 1:**
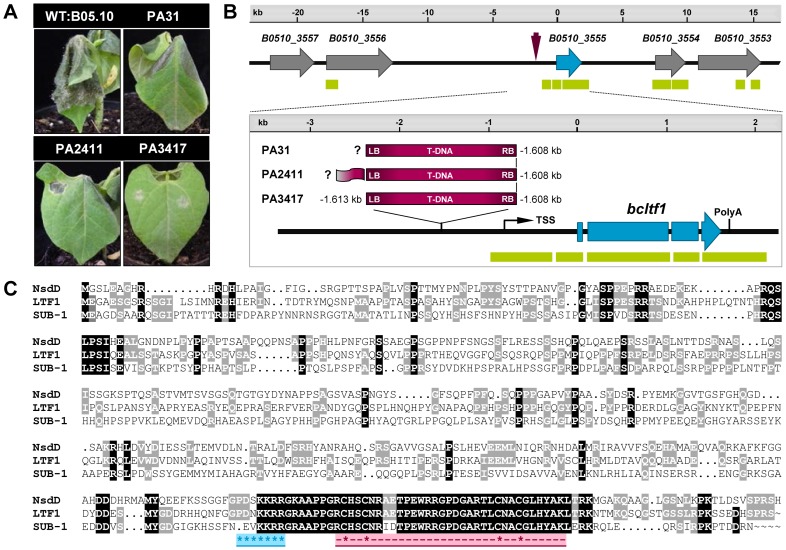
Identification of *bcltf1* as putative virulence factor by an ATMT approach. (**A**) Virulence phenotypes of the recipient strain B05.10 and the ATMT mutants PA31, PA2411, and PA3411. Primary leaves of *Phaseolus vulgaris* were inoculated with conidial suspensions and incubated for 5 d. (**B**) T-DNA insertion sites in the ATMT mutants were identified by TAIL-PCR analyses. In all three mutants, the insertion of the T-DNA (purple arrow) took place 1.608 kb upstream of an ORF hereinafter referred to as *bcltf1* (blue arrows). The genomic region in strain B05.10 (*B. cinerea* Database, Broad Institute) with annotated genes and matching EST sequences (green bars) is shown. (**C**) Sequence alignment of BcLTF1 with homologous proteins from *A. nidulans* and *N. crassa*. The alignment was generated using ClustalW (http://genius.embnet.dkfz-heidelberg.de/) and the following protein sequences: *B. cinerea* LTF1 (481 aa; B0510_3555), *A. nidulans* NsdD (461 aa; AAB16914), and *N. crassa* SUB-1 (466 aa; NCU01154.5). Amino acids that are identical in all sequences are shaded black, amino acids that are shared by *B. cinerea* and *N. crassa* or *A. nidulans* are shaded in gray. The nuclear localization signal (NLS) is highlighted in blue, the GATA zinc finger motif (PF00320) with the four conserved cysteine residues (indicated by asterisks) is highlighted in pink.

To identify genes in *B. cinerea* that are associated with the infection process, 2,367 hygromycin-resistant mutants obtained through *A. tumefaciens*-mediated transformation (ATMT) were screened for virulence on detached tomato leaves. The assay revealed 560 mutants that were either avirulent or less virulent than the parental wild-type strain B05.10 [39]. So far, phenotypes have been confirmed for 193 mutants on primary leaves of living bean plants (*Phaseolus vulgaris*), including the mutants PA31, PA2411, and PA3417 (Fig. 1A). These mutants are impaired in their ability to penetrate (PA3417) or to colonize the host tissue (PA31, PA2411), and are furthermore affected in light-dependent differentiation (data not shown). TAIL-(thermal asymmetric interlaced) PCR analyses revealed that the T-DNA integrations in the genomes of the mutants have occurred in the same genomic region (Fig. 1B). Identical integration events occurred for the RB flanks of the T-DNAs in all mutants i.e. 1.608 kb upstream of the annotated open reading frame (ORF) B0510_3555 (Broad Institute; for details see Materials and Methods), while different situations were found for the LB-flanking regions. In PA3417, the T-DNA insertion caused the deletion of four base pairs, in the other two mutants TAIL-PCR failed because no amplicons were obtained (PA31), or the remaining vector sequence was amplified (PA2411).

BlastP analyses using the sequence of B0510_3555 as query revealed the GATA-type transcription factors NsdD and SUB-1 from *A. nidulans* (e-value 6e-46) and *N. crassa* (e-value 9e-40), respectively, as putative orthologues. The alignment of protein sequences of B0510_3555, NsdD and SUB-1 showed low levels of sequence similarity (48% and 35%, respectively), except for the highly conserved GATA zinc finger domains in the C-terminal regions (Fig. 1C). Due to its expression profile and functions in *B. cinerea* - as described in the following - the protein is referred to as BcLTF1 (***B*** *. cinerea* **l**ight-responsive **t**ranscription **f**actor 1).

To confirm the linkage between the identified T-DNA integrations upstream of *bcltf1* and the observed phenotypes of PA31, PA2411, and PA3417, *bcltf1* was deleted and over-expressed in the B05.10 genomic background. Two different replacement mutants exhibiting identical phenotypes were generated by deleting either the ORF only (Δ*bcltf1*-A6) or the ORF including 1.7-kb of the 5′-noncoding region (Δ*bcltf1*-B1). Expression of *bcltf1* under control of a constitutive promoter (P*oliC*) in the wild-type background resulted in two over-expressing mutants (OE::*bcltf1*-T6 and -T7). Complementation of deletion mutants was done by re-introduction of *bcltf1* into the native gene locus (*bcltf1* ^COM^) (for details, see Materials and Methods, Table 1, Fig. S1, Table S1).

**Table 1:** *B. cinerea* strains used in this study.

Strain	Genotype	Reference
**WT:B05.10**	Isolate from *Vitis vinifera* (Germany); *MAT1-1*	[14]
**WT:SAS405**	Isolate from *Vitis vinifera* (Italy); *MAT1-2*	[12]
**PA31, PA2411, PA3417**	B05.10, T-DNA::*hph*, ∼1.6 kb upstream of *bcltf1*, heterokaryon	[39]
**Δ** ***bcltf1*** **-A** [(A1), A6]	B05.10, Δ*bcltf1::hph*, homokaryon	This study
**Δ** ***bcltf1*** **-B** [B1]	B05.10, Δ*bcltf1 including 5′-noncoding region::hph*, homokaryon	This study
***bcltf1*** **^COM^** [T2, T3]	B05.10, Δ*bcltf1::hph, bcltf1::nat1* in loco, heterokaryon	This study
**OE::** ***bcltf1*** [T6, T7]	B05.10, P*oliC::bcltf1-gfp::nat1* in *bcniiA*, heterokaryon	This study
**Δ** ***bcltf1*** **+P** ***oliC*** **::** ***bcltf1-gfp*** [T3, T4]	B05.10, Δ*bcltf1::hph*, P*oliC::bcltf1-gfp::nat1* in *bcniiA*, heterokaryon	This study
**Δ** ***bcltf1*** **+P** ***bcltf1*** **::** ***bcltf1-gfp*** [T2, T5]	B05.10, Δ*bcltf1::hph, bcltf1-gfp::nat1* in loco, heterokaryon	This study
**WT+** ***grx-rogfp2***	B05.10, P*oliC*::*rogfp2::hph* in *bcniiA*, heterokaryon	[45]
**Δ** ***bcltf1*** **+** ***grx-rogfp2*** [T2, T3]	B05.10, Δ*bcltf1::hph*, P*oliC*::*rogfp2::nat1* in *bcniiA*, heterokaryon	This study

### *Bcltf1* expression is strongly induced by light

**Figure 2:**
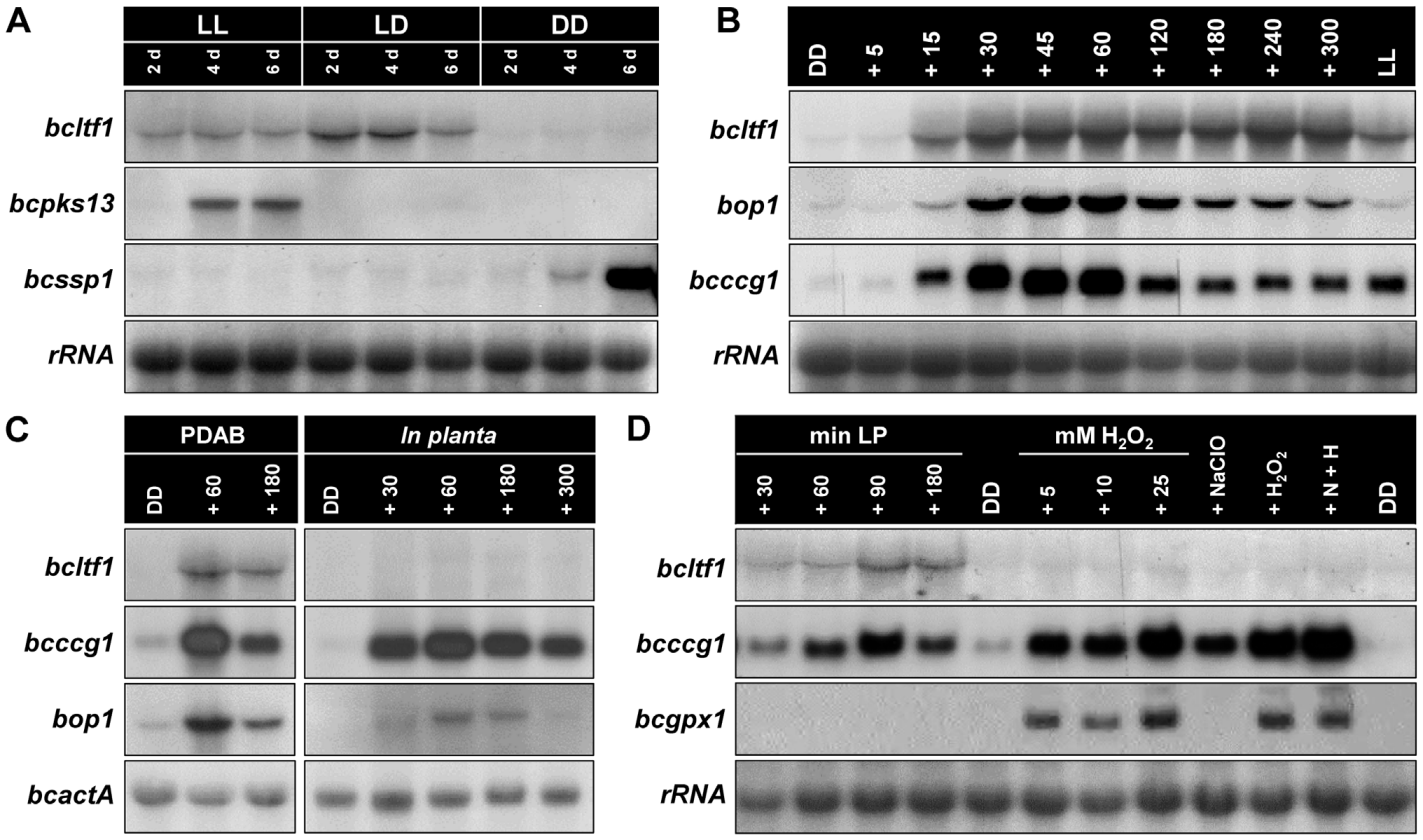
Expression of *bcltf1* depends on light. Gene expression was studied in wild-type strain B05.10 by northern blot analyses. rRNA or *bcactA* are shown as loading controls. For more details see Materials & Methods. (**A**) *Bcltf1* is expressed during conidiation but not during sclerotial development. B05.10 was incubated on solid CM for the indicated time periods in continuous light (LL) and light-dark (LD) for induction of conidiation, and in continuous darkness (DD) for induction of sclerotium formation (sclerotia initials at 6 dpi). Genes exhibiting stage-specific expression profiles are shown: expression of *bcpks13* is restricted to premature conidiation in LL, *bcssp1* is exclusively expressed in developing sclerotia. (**B**) Expression of *bcltf1* is induced by light. Wild type was incubated on solid CM for 52 h in DD or LL. DD-grown cultures were then exposed to white light for the indicated periods (5 to 300 min). (**C**) *Bcltf1* is weakly expressed during infection of *P. vulgaris*. Primary leaves of living plants were inoculated with conidial suspensions of the wild type (four droplets per leaf) and incubated for 3 d in DD. Then plants were exposed to light for the indicated periods (30 to 300 min) or kept in DD. For comparison, light responses of B05.10 grown on solid PDAB (containing mashed bean leaves) are shown. (**D**) Expression of *bcltf1* does not respond to oxidative stress. Liquid CM was inoculated with non-sporulating mycelia, and incubated for 3 d in DD at 170 rpm. Then, cultures were harvested (DD, controls), exposed to light (30 to 180 min), or exposed in darkness to oxidative stress induced by H_2_O_2_ (5 to 25 mM) or singlet oxygen (generated by simultaneous addition of 10 mM NaClO and 10 mM H_2_O_2_ (N+H)). All cultures that were exposed to oxidative stress were harvested after 30 min. *Bcgpx1* is a known H_2_O_2_-induced gene.

Given that BcLTF1 is implicated in regulation of light-dependent differentiation as suggested by the ATMT phenotypes, we studied its expression pattern during conditions inducing either conidiation (incubation in continuous light (LL) or in 12 h light/12 h dark rhythm (LD)) or sclerotia formation (incubation in continuous darkness (DD)) in the wild-type strain B05.10. Northern blot analyses revealed that *bcltf1* was highly expressed during incubation in the light while only very weak expression occurred during sclerotial development in the dark. For comparison, expression of *bcssp1* (homologue of *S. sclerotiorum* “*sclerotia-specific protein 1*” [40]) is restricted to developing sclerotia while expression of *bcpks13* (polyketide synthase (PKS) putatively involved in melanin biosynthesis [41]) is restricted to conidiation in LL (Fig. 2A). To further analyze the effect of light on the expression of *bcltf1*, we exposed wild-type cultures that were grown for 2 d in DD for different periods to white light (5 to 300 min). High expression levels of *bcltf1* became visible from 15 min light pulse (LP) while only low expression occurred in cultures that were kept in DD. In contrast to other light-induced genes such as *bop1* (opsin-like protein [30]) and *bcccg1* (homologue of *N. crassa* “*clock-controlled gene-1*” [42]) whose expression levels decreased again after prolonged exposure to light (from exposure times of 120 min and longer), expression levels of *bcltf1* remained stable (Fig. 2B). Light induction of *bcltf1*, *bop1*, and *bcccg1* was also observed during infection of *P. vulgaris* leaves (in stage of lesion spreading at 3 dpi), even though expression levels of *bcltf1* were very low in comparison to those found in cultures grown on solid PDAB medium made from mashed bean leaves (Fig. 2C). Finally, dark-grown submerged cultures of B05.10 were exposed to ROS under dark conditions. Exposure to light, but neither the addition of hydrogen peroxide (H_2_O_2_), nor the presence of singlet oxygen (^1^O_2_), superoxide anions (O_2_−•) and hydroxyl radicals (OH•) affected the expression of *bcltf1*. Expression levels of *bcccg1* increased in response to all treatments in contrast to *bcgpx1* (encoding a glutathione peroxidase) that is specifically induced by H_2_O_2_ (Fig. 2D).

Taken together, among the conditions tested *bcltf1* is exclusively highly expressed in response to light, and represents therefore an eligible candidate for controlling differentiation processes (conidiation vs. sclerotial development) and virulence in a light-dependent manner.

### BcLTF1 is localized in the nuclei

**Figure 3:**
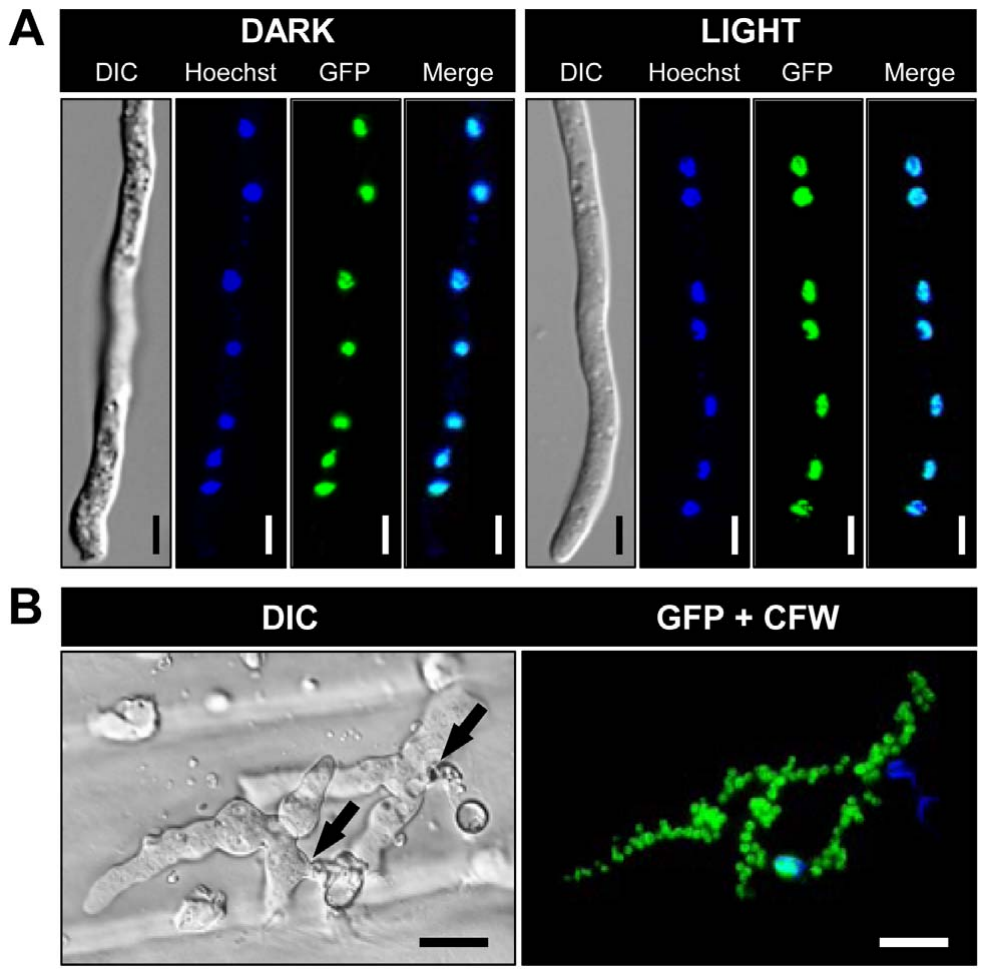
BcLTF1-GFP fusion proteins are localized in the nuclei. (**A**) Subcellular localization of BcLTF1-GFP does not depend on illumination conditions. The fusion protein was expressed from the constitutive *A. nidulans oliC* promoter in the Δ*bcltf1* background. Conidia were incubated on microscope slides for 18 h in DD (dark), or 12 h in DD followed by 6 h in LL (light). Nuclei were visualized using the fluorescent dye Hoechst 33342. Scale bars, 5 µm. (**B**) BcLTF1-GFP localizes to the numerous nuclei in infectious hyphae. Onion epidermis strips were inoculated with conidial suspensions and incubated for 24 h in DD. Conidia and hyphae on the surface were stained with the fluorescent dye calcofluor white (CFW). Sites of penetration are indicated by black arrows. Scale bars, 20 µm.

To localize BcLTF1 in living hyphae, the coding sequence of *bcltf1* was fused to the codon-optimized gene encoding the green fluorescent protein (GFP) [43] and expressed under control of a constitutive promoter in the *bcltf1* deletion mutant (Δ*bcltf1*+P*oliC*::*bcltf1-gfp*) (for details, see Materials and methods, Table 1, Fig. S1E). Merged images of GFP fluorescence and nucleic acid-specific Hoechst staining patterns showed that BcLTF1-GFP localizes to the nuclei in germinated conidia irrespective of the applied light conditions (Fig. 3A). Nuclear localization of BcLTF1-GFP was also observed in infectious hyphae during infection of onion epidermal cells (Fig. 3B). Similar results were obtained for BcLTF1-GFP that was expressed from its native promoter (Δ*bcltf1*+P*bcltf1*::*bcltf1-gfp*). GFP fluorescence, although weaker when compared to that of the constitutively expressed fusion protein, was detected in the nuclei independently of the illumination treatment (data not shown). Expression of BcLTF1-GFP under control of P*oliC* or its native promoter in the Δ*bcltf1* genomic background indicated the functionality of the fusion protein, as the native expression rescued all phenotypes of the deletion mutant while P*oliC*-driven expression resulted in phenotypes that are presumably due to the inappropriate expression of BcLFT1 especially in dark conditions (Fig. S2, Fig. S1F). Accordingly, mutants expressing P*oliC::bcltf1-gfp* in the wild-type background were generated as BcLTF1-overexpressing strains hereafter referred to OE::*bcltf1* (T6, T7) (Fig. S1F, Table 1).

### *Bcltf1* mutants are affected in light-dependent differentiation programs

**Figure 4:**
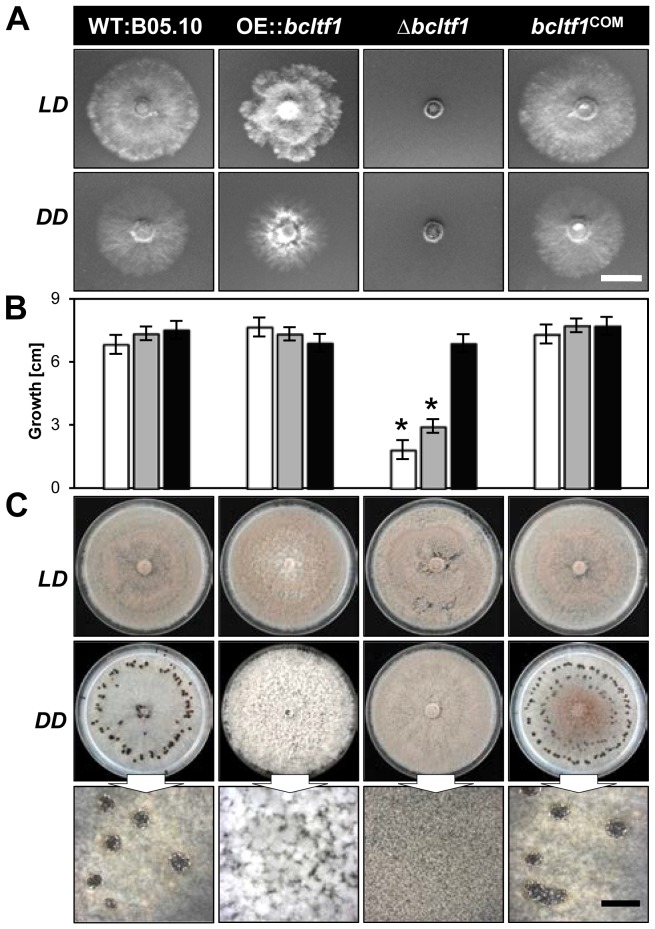
Mutations of *bcltf1* alter light-dependent growth and differentiation programs. (**A**) BcLTF1 is essential for allowing growth on minimal medium. Indicated strains were incubated for 2 d on solid CD. Scale bar, 1 cm. (**B**) Exposure to light negatively affects radial growth rates of Δ*bcltf1* mutants on synthetic complete medium. Strains were incubated on solid CM in LL (white bars), LD (gray bars), and DD (black bars). Colony diameters (growth in cm) were determined after 3 d of incubation. Mean values and standard deviations were calculated from five colonies per strain and light condition. Asterisks indicate significant differences compared to WT:B05.10 in each condition (p<0.001). (**C**) Both overexpression and deletion of BcLTF1 modulate the differentiation program in DD. Strains were incubated for 14 d on solid CM. Close-up views of the cultures incubated in DD are shown in the lower panel. Scale bar, 5 mm.

The deletion as well as the over-expression of *bcltf1* cause severe growth phenotypes that are related to the light treatment. Deletion mutants (Δ*bcltf1*-A6, -B1) failed to grow on minimal medium (CD) in any light condition (Fig. 4A), and radial growth rates on synthetic complete medium (CM) correlated with the exposure times to light: while growth rates nearly equivalent to the wild type were observed in DD (92% of WT), incubation in LD and LL conditions significantly decreased growth rates to 43% and 27% of DD, respectively (Fig. 4B). The inhibitory effect of white light could be assigned to the blue light fraction, as growth rates were similarly decreased under incubation in continuous white and blue light, while growth rates in green, yellow and red light were comparable to those of Δ*bcltf1* cultures grown in DD (Fig. S3). Although Δ*bcltf1* mutants were severely impaired in growth in LD, they colonized the whole Petri dish after two weeks of incubation accompanied by reduced aerial hyphae formation and precocious and enhanced conidiation (155% of conidia produced by WT; data not shown). In contrast, the overexpressing mutants produced more aerial mycelia and reduced numbers of conidia giving the colonies a fluffy appearance. Light of different wavelengths (in LD rhythm) failed to alter the phenotypes of Δ*bcltf1* and OE::*bcltf1* mutants. In contrast, the wild type formed more aerial hyphae and fewer conidia and sclerotia during incubation in blue and yellow/red light, respectively (data not shown). In general, phenotypes of the dark-grown cultures were more pronounced than those incubated in LD as both Δ*bcltf1* and OE::*bcltf1* mutants failed to produce any sclerotia. Instead, the deletion mutants produced conidia and the overexpression mutants accumulated cotton ball-like mycelial aggregates in the dark (Fig. 4C).

Evidently, light and BcLTF1 have an impact on colony morphology and the mode of reproduction, however, other differentiation programs in *B. cinerea* are not affected by light and mutations of *bclft1*. Conidial germination is induced by nutrients or hydrophobic surfaces in a similar fashion in wild type, OE::*bcltf1* and Δ*bcltf1* mutants in LL and DD, and germling fusions via conidial anastomosis tubes (CATs) were observed for all strains (Fig. S4A; data not shown). Nevertheless, the forced expression of *bcltf1* that is marked by bright nuclear BcLTF1-GFP signals, results in malformed germ tubes (Fig. S4B). The lack of the GFP signal accompanied by a wild-type-like morphology in a percentage of germ tubes suggests that the overexpression construct might become lost during conidiogenesis, so the outcome of studies involving OE::*bcltf1* conidia needs to be carefully considered.

### Moderate expression levels of *bcltf1* are crucial for virulence

**Figure 5:**
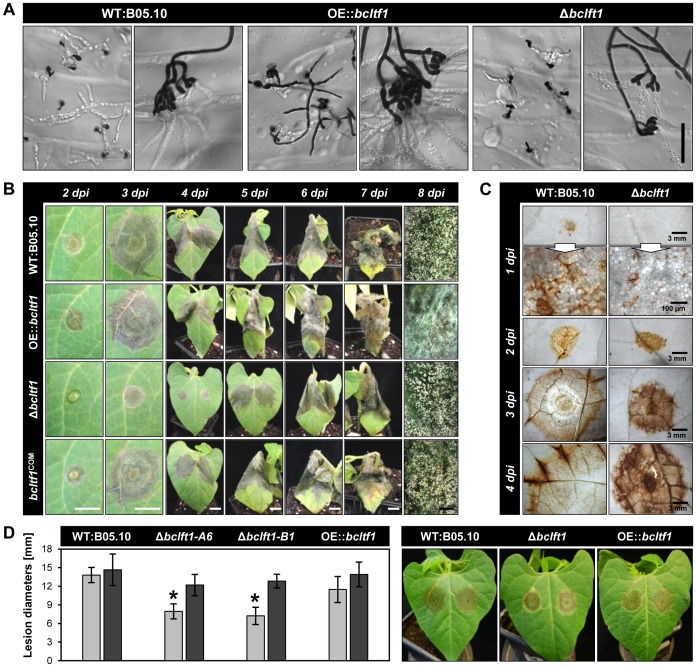
BcLTF1 is required for full virulence. (**A**) Overexpression of *bcltf1* affects the formation of infection structures. Onion epidermal strips were inoculated with conidial suspensions (on the left) or non-sporulating mycelia (on the right), and incubated for 24 h in DD. Conidia and hyphae on the surface were stained with lactophenol blue whereas invasively growing hyphae remain colorless. Scale bars, 50 µm. (**B**) Deletion of *bcltf1* impairs the capability to colonize primary leaves of *P. vulgaris*. Leaves of living plants were inoculated with conidial suspensions and incubated in LD. White scale bars, 1 cm; black scale bars, 2 mm. (**C**) Spreading lesions provoked by Δ*bcltf1* mutants are characterized by increased H_2_O_2_ accumulation. Primary leaves were inoculated with conidial suspensions, detached 1 to 4 dpi, and incubated for 2 h in DAB solution. Plant tissues were decolorized prior to microscopy. (**D**) Addition of ascorbic acid restores virulence of Δ*bcltf1* mutants. Conidia were suspended in GB5 (gray) or in GB5 supplemented with 5 g/l ascorbic acid (black). Mean values and standard deviations of lesion diameters were calculated from eight lesions per strain and condition at 3 dpi. Asterisks indicate significant differences compared to WT:B05.10 in each condition (p<0.001). Pictures of lesions derived from ascorbic acid-supplemented conidial suspensions were taken 3 dpi.

Though all T-DNA insertions upstream of *bcltf1* impair virulence, differences exist between mutant PA3417, that is not able to penetrate resulting in the accumulation of mycelia on the top of the leaf, and mutants PA31 and PA2411 that are impaired in their ability to colonize the host tissue (Fig. 1A). However, the penetration defect of PA3417 is restricted to the conidia as infections from mycelia have been observed (data not shown). To examine how BcLTF1 is implicated in the infection process, the defined mutants (Δ*bcltf1*, OE::*bcltf1*) were tested for their capabilities to penetrate and to proliferate inside a living host. To monitor the penetration events, onion epidermal strips were inoculated with conidial suspensions or agar plugs with non-sporulating mycelia (Fig. 5A). At 24 hpi, all strains had formed branched infection structures (infection cushions) originated from the already established mycelia on the agar plugs. Presumably, OE::*bcltf1* mutants produced them more frequently than the wild type due to the significantly increased proliferation of aerial hyphae. In contrast, infection cushions were less often observed for Δ*bcltf1* mutants known to form fewer aerial hyphae (data not shown). Conidia derived from wild-type and Δ*bcltf1* strains formed short germ tubes that immediately penetrated the host cells indicating that the transcription factor is dispensable for entering the host. However, the majority of conidia with elevated *bcltf1* expression levels produced elongated and branched germ tubes that failed to penetrate.

Primary leaves of living bean plants (*P. vulgaris*) were inoculated with conidial suspensions or mycelia-containing agar plugs derived from wild-type and mutant strains, and incubated in LD and DD to study the impact of light on infection. As can be seen from the time course experiments, Δ*bcltf1* mutants were significantly impaired in the infection process irrespective of the inoculation method and incubation conditions (in DD or LD) because in all cases distinct primary lesions emerged one day later than for the wild type and the OE::*bcltf1* mutants (Fig. 5B, Fig. S5A,B). The reduced proliferation of Δ*bcltf1* hyphae inside the host became visible by 1 dpi in onion cells, and was likewise visualized in living bean tissues by trypan blue staining at 2 and 3 dpi (Fig. S5C). However, infection by the deletion mutant progressed until the whole leaf was colonized (9 dpi) and conidia were formed. Although germ tubes of OE::*bcltf1* were severely impaired in their capacity to penetrate onion epidermal cells, infections of *P. vulgaris* derived from conidia and mycelia were comparable to those caused by the wild type with regard to lesion expansion. In contrast, colonization of the host tissue by OE::*bcltf1* was accompanied by increased accumulation of aerial hyphae and reduced conidiation (Fig. 5B, Fig. S5A). In summary, light or its absence did not affect the infection process by wild type and *bcltf1* mutant strains, and elevated and reduced expression levels of *bcltf1* resulted in reduced penetration rates via germ tubes (OE::*bcltf1*) and retarded infection (Δ*bcltf1*), respectively.

### Increased ROS accumulation accounts for reduced virulence of Δ*bcltf1* mutants

*B. cinerea* induces an oxidative burst and hypersensitive cell death in the host, and it was shown that the degree of virulence on *Arabidopsis* correlates with the levels of superoxide (O_2_−) and hydrogen peroxide (H_2_O_2_) [44]. To monitor the accumulation of H_2_O_2_ in wild type- and Δ*bcltf1*-infected plant tissues, inoculated bean leaves were sampled at 1 to 4 dpi and treated with 3,3′-diaminobenzidine (DAB); a compound that is oxidized by H_2_O_2_ in the presence of peroxidases yielding a dark-brown product. Microscopic observations revealed H_2_O_2_ accumulation in anticlinal walls of epidermal cells that were in close contact with wild-type germlings at 1 dpi. In contrast, no H_2_O_2_ accumulation was observed beneath the Δ*bcltf1* infection droplets at this time point even though conidia had germinated and entered the plant tissue (data not shown). However, after a further two days, significantly increased H_2_O_2_ accumulation in spreading lesions of the *bcltf1* deletion mutant was observed (Fig. 5C). Therefore, in this particular case, increased ROS accumulation does not positively correlate with virulence. To explore the possibility that the elevated ROS levels may negatively affect the ability of the Δ*bcltf1* mutant to colonize the host tissue, we added ascorbic acid as an antioxidant to the conidial suspensions of wild type and *bcltf1* mutants prior to inoculation. Whereas no differences were observed for infections by wild type and OE::*bcltf1* strains, the addition of the antioxidant restored virulence of Δ*bcltf1* mutants as deduced from lesion sizes at 3 dpi (Fig. 5D). Consequently, the abnormal ROS accumulation may account for the virulence defect of the Δ*bcltf1* mutants.

### BcLTF1 is required for maintaining ROS homoeostasis

**Figure 6:**
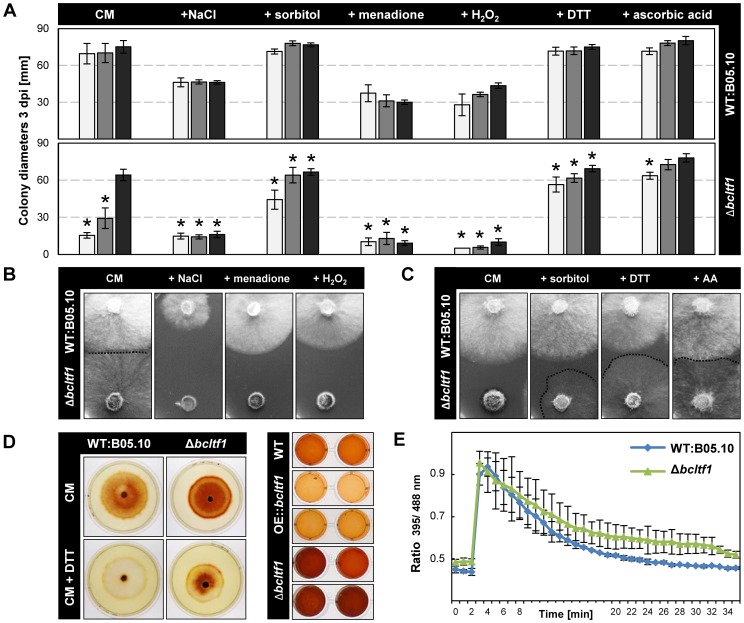
BcLTF1 is needed to cope with oxidative stress and regulates the generation of ROS. (**A**) Radial growth rates of Δ*bcltf1* mutants are affected by supplements. Strains were incubated in LL (white), LD (gray), or DD (black) on solid CM (control), and CM with 0.7 M NaCl, 0.7 M sorbitol, 300 µM menadione, 7.5 mM H_2_O_2_, 800 µM DTT (dithiothreitol) or 28 mM AA (ascorbic acid). Mean values and standard deviations were calculated from five colonies per strain and condition. Asterisks indicate significant differences compared to WT:B05.10 in each condition (p<0.001). (**B**) Salt and oxidative stress prevent growth of Δ*bcltf1* mutants even in the absence of light. Pictures were taken after 2 d in DD. (**C**) Ascorbic acid and DTT restore light tolerance but not the proliferation of aerial hyphae. Pictures were taken after 2 d in LL. Hardly visible Δ*bcltf1* colonies are highlighted by dashed lines. (**D**) *Bcltf1* mutants are affected in generation of H_2_O_2_. Strains were incubated for 2 d in DD on CM or CM supplemented with 750 µM DTT. Then, colonies were flooded with DAB solution. DTT prevents oxidation of DAB in the range of wild-type colonies but not in the range of Δ*bcltf1* colonies (on the left). Identical quantities of fresh mycelia of B05.10, Δ*bcltf1* (A6, B1) and OE::*bcltf1* (T6, T7) were incubated in DAB solution (on the right). (**E**) The cytosolic glutathione redox potential is not significantly affected in the absence of *bcltf1*. Reactions of redox-sensitive reporter roGFP2 expressed in Δ*bcltf1* and wild type were compared. Reduction of roGFP2 is displayed after an oxidation event provoked by addition of 10 mM H_2_O_2_ after 2 min. Indicated values are the means of triplicates; standard deviations are shown. For details, see Materials and Methods.

For reasons thus far un-established, BcLTF1 is required to tolerate light during saprophytic growth on solid media, but not during plant colonization. To unravel the mechanism of light sensitivity, we modified the standard growth medium with different supplements and monitored colony growth and morphology during incubation in LL, LD and DD. Osmotic stabilization of the medium by adding 0.7 M sorbitol elevated growth rates of Δ*bcltf1* mutants in LL and LD conditions almost to those found in DD (66% and 94% of DD) (Fig. 6A) suggesting that the mutants have difficulty to cope with hypo-osmotic conditions, especially during illumination. On the other hand, the addition of 0.7 M NaCl appeared to cause ionic stress and prevented growth of Δ*bcltf1* mutants also during incubation in DD (25% of DD). These findings may imply that cell wall integrity and/or the osmotic stress response is impaired when *bcltf1* is absent.

Considering the finding of the altered ROS accumulation in Δ*bcltf1*-infected plant tissues and the compensating effect of ascorbic acid in virulence, we pursued two strategies to study whether ROS play a role in the light-dependent growth defect of the deletion mutants. Firstly, the effect of oxidative stress caused by addition of 300 µM menadione (artificial source of superoxide radicals (O^−^_2_•)) and 7.5 µM H_2_O_2_ (a source of highly reactive hydroxyl radicals (OH•) upon reaction with metal ions), respectively, was monitored revealing that the deletion mutants were not able to cope with these conditions irrespective of the light conditions (Fig. 6A,B). Secondly, the use of antioxidants was intended to protect fungal strains against ROS. Both the addition of 800 µM dithiothreitol (DTT) and 28 mM ascorbic acid restored radial growth rates of Δ*bcltf1* mutants in LL and LD, but not the proliferation of aerial hyphae (Fig. 6C). To study whether the growth defect of the Δ*bcltf1* mutants on minimal medium (CD) can be rescued by reducing oxidative stress, we supplemented CD medium containing either nitrate or ammonium a single nitrogen source with ascorbic acid. In fact, the supplementation increased the growth rates of the mutants to certain extents in LD and DD, but failed to restore wild-type growth rates (Fig. S6). The fact that Δ*bcltf1* mutants are hypersensitive to exogenously applied oxidative stress even in the absence of light, and that on the contrary, the supplementation with antioxidants facilitated growth during incubation in LL indicate that the deletion mutants are unable to cope with oxidative stress that arises during the exposure to light.

To detect ROS production by the different strains, colonies of wild type and *bclft1* mutants grown on CM and DTT-supplemented CM were stained with DAB solution. The intensive brown coloration of mutant colonies, even in presence of DTT that creates a reducing environment, indicated an increased accumulation of H_2_O_2_ in the range of the Δ*bcltf1* colonies in comparison to the wild type (Fig. 6D). In a second approach, the H_2_O_2_ production by equal amounts of biomasses of wild type, *bcltf1* deletion and overexpressing mutants was studied, confirming the increased H_2_O_2_ production by the deletion mutants and revealing furthermore the decreased production by mutants overexpressing BcLTF1 (Fig. 6D). Formation of O^−^_2_• was monitored by treating wild type and mutant mycelia with nitro blue tetrazolium (NBT). Blue precipitates were found in hyphal tip segments of all strains (data not shown) demonstrating that mutations of *bcltf1* do not alter the net accumulation of O_2_^−^. The fact that Δ*bcltf1* mutants accumulate higher amounts of H_2_O_2_ under all light conditions, either caused by its inappropriate production and/or detoxification, may explain the general hypersensitivity of the mutants to (additional) exogenously applied oxidative stresses such as the exposure to menadione, H_2_O_2_ or light.

To see whether the cellular redox status (glutathione pool) is altered in Δ*bcltf1* mutants, we expressed the redox-sensitive green fluorescent protein (roGFP2) in the mutant background and compared the ratio of fluorescence intensities (395 nm = oxidized state/488 nm = reduced state) with those of the wild type expressing roGFP2 [45]. Similar initial redox statuses were found in Δ*bcltf1* and wild-type hyphae, and roGFP2 was oxidized and subsequently reduced in a similar fashion in both strains after addition of 10 mM H_2_O_2_ (Fig. 6E). Thus, the altered ROS homoeostasis of the mutants that is characterized by the overproduction of H_2_O_2_ and the hypersensitivity to oxidative stress and light is not accompanied by an altered cytosolic redox status.

### BcLTF1 modulates the transcriptional responses to light

To gain broader insight into the functions of BcLTF1 especially in those that are related to illumination, a genome-wide approach was initiated to compare gene expression profiles in the wild type and the Δ*bcltf1* mutant in response to a light stimulus. For these experiments, strains were cultivated in DD, and then exposed to white light for 60 min (WT:B05.10+LP; Δ*bcltf1*+LP) or kept in darkness for additional 60 min (WT:B05.10-D; Δ*bcltf1*-D) (for details, see Material and Methods). RNA from four biological replicates was extracted, labeled and hybridized to NimbleGen microarrays containing oligonucleotides representing all predicted *B. cinerea* genes as well as non-matching expressed sequence tags (ESTs) [2].

**Figure 7:**
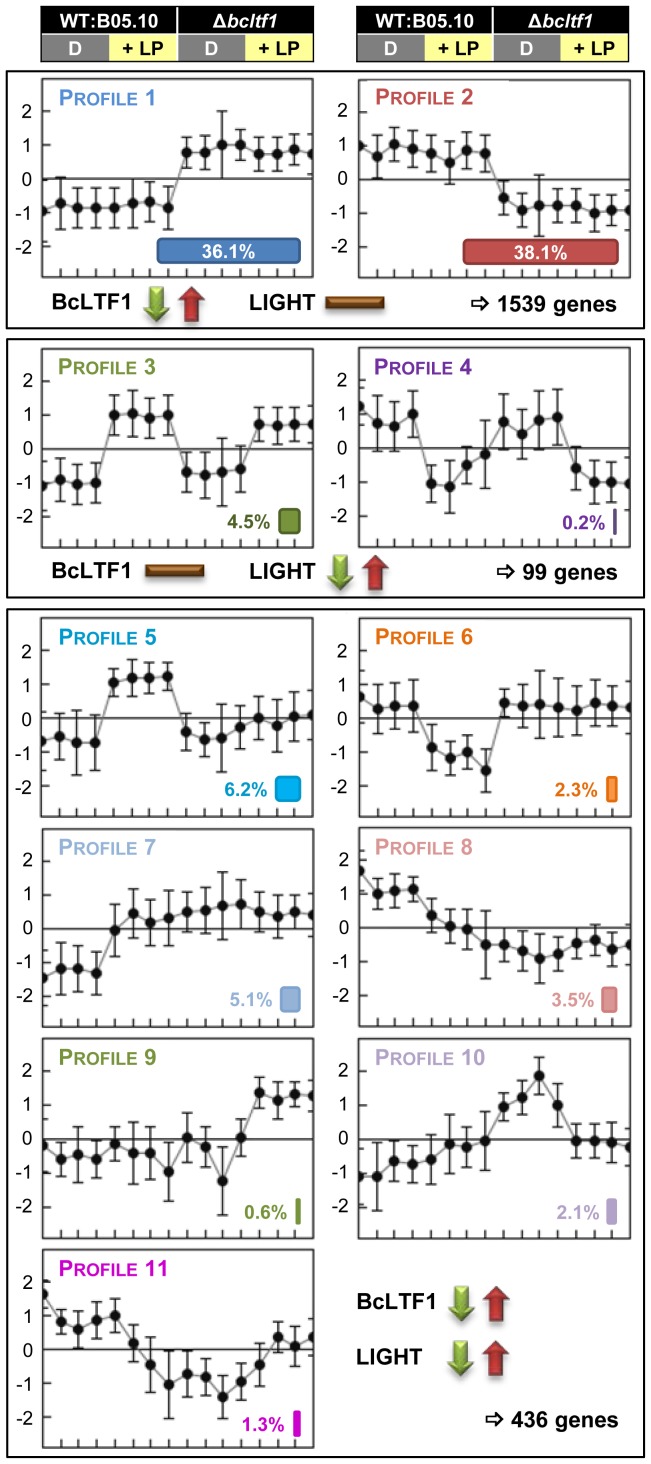
BcLTF1 modulates the transcriptional responses to light. Wild type B05.10 and the Δ*bcltf1* mutant were grown on solid CM for 52 h in DD; then, cultures were exposed for 60 min to light (+LP) or kept for additional 60 min in darkness (D). Four biological replicates were performed. RNA samples were labeled and hybridized to microarrays with all predicted genes of *B. cinerea*. Statistical analysis revealed 2,074 differentially expressed genes. Relative expressions of the genes (i.e. log2-normalized intensities scaled by gene) in the four conditions (16 hybridizations) were clustered, and 11 of the 14 theoretically possible expression profiles were found (for more details, see Materials and Methods). This figure represents the centroid views of the 11 expression profiles (x = hybridization and y = log2-normalized intensity scaled by gene). The percentages among the 2,074 genes are indicated for each profile. Genes whose expression is only affected by the deletion of *bcltf1* belong to profiles 1 (748 genes) and 2 (791 genes). Profiles 3 (94 genes) and 4 (5 genes) comprise genes whose expression is affected by light in a similar manner in both genomic backgrounds (WT, Δ*bcltf1*). Genes (436) belonging to the other profiles are affected by light treatment and the deletion of *bcltf1*.

Statistical tests were performed to detect differentially expressed genes: light responses in wild type (+LP/D in WT) and mutant (+LP/D in Δ*bcltf1*), and Δ*bcltf1* effects in darkness (Δ*bcltf1*/WT in D) and light (Δ*bcltf1*/WT in LP). In total, 2,156 differentially expressed spotted genes corresponding to 2,074 distinct genes were identified and could be assigned to 11 expression profiles (Fig. 7, Table S2). 293 genes showed a light-responsive pattern in the wild-type background: the expression of 244 genes was induced by light (referred to as light-induced genes; *bclig1-244*), and the expression of 49 genes was repressed by light (referred to as light-repressed genes; *bclrg1-49*). The expression levels of the majority of light-responsive genes were altered in the absence of BcLTF1, i.e. light induction/repression did not happen (genes in profiles 5 and 6) or just disappeared as genes are already de-regulated in Δ*bcltf1* in the absence of light (genes in profiles 7 and 8). Only 34% of light-responsive genes (in profiles 3 and 4) exhibited similar light responses in both genomic backgrounds, demonstrating that the absence of *bcltf1*, which itself is responsive to light, severely affects the expression pattern of other light-responsive genes. Interestingly, some genes were found whose expression responded to light exclusively in the absence of BcLTF1; they were either induced (12 genes in profile 9) or repressed by light (44 genes in profile 10). Nevertheless, the loss of the BcLTF1 had a more pronounced effect on genome-wide gene expression than the light stimulus. In total, the expression levels of 1,972 spotted genes corresponding to 1,905 distinct genes were changed in the Δ*bcltf1* mutant and most of them (1,539 genes; 81%) were not related to a light response: 748 genes were overexpressed (profile 1) and 791 genes were underexpressed (profile 2) irrespective of the illumination condition (Fig. 7, Table S2).

Functional enrichment analyses using the GSEA (Gene Set Enrichment Analyses [46]–[47]) method and defined sets of genes were performed to reveal biological processes that are affected by light and/or in the absence of BcLTF1. Hence, the group of light-induced genes in wild type is enriched for genes implicated in the oxidative stress response (OSR) or light perception, while amino acid-transporter-encoding genes are overrepresented in the group of light-repressed genes. Even though a majority of *bcltf1*-dependent genes display similar expression profiles in darkness and light, the group of overexpressed genes in the *Δbcltf1* mutant in darkness is significantly enriched in genes involved in light perception and respiration, and the group of overexpressed genes in the light is enriched in secondary metabolism-related genes. In both light conditions, the group of underexpressed genes in Δ*bcltf1* is enriched in transporter-encoding genes (Table 2).

**Table 2:** Functional categories that are enriched due to light treatment or deletion of BcLTF1.

	WT:B05.10		Δ*bcltf1*		In darkness (D)		+light pulse (LP)	
Name of gene set (size)	UP+LP	DOWN+LP	UP+LP	DOWN+LP	UP Δ*bcltf1*	DOWN Δ*bcltf1*	UP Δ*bcltf1*	DOWN Δ*bcltf1*
**Oxidative stress response** (OSR) (87)	**1.65** (<0.001)							
**Photoreceptors** (10)	**1.52** (<0.001)		**1.57** (<0.001)		**1.75** (<0.001)			
**Primary metabolism – respiration** (69)					**1.67** (0.02)			
**Alternative respiration** (15)					**1.58** (<0.001)			
**Secondary metabolism** (227)							**1.50** (<0.001)	
**Transporters** (393)				**1.38** (0.03)		**1.61** (<0.001)		**1.46** (0.03)
**Amino acid transporters** (48)		**1.84** (<0.001)		**1.55** (0.03)		**1.90** (<0.001)		
**MFS-type sugar transporters** (68)				**1.56** (0.03)				**1.64** (0.03)
**Mitochondrial carrier proteins** (37)					**1.52** (0.04)			
**P-type ATPases transporters** (22)					**1.45** (0.04)			

Gene sets were as previously defined by Amselem et al. [2] or defined in the present study. GSEA (http://www.broadinstitute.org/gsea/) toolkit was used to determine whether gene sets show statistically significant, concordant differences due either to light treatment or BcLTF1 deletion. Normalized Enrichment Scores (NES) and p-values (in brackets) are indicated for cases where p-values <0.05.

### Identification of putative regulators of light-dependent differentiation

Currently, it is not known how light and its absence, trigger differentiation in *B. cinerea*. Because young undifferentiated mycelia of the wild type, that are able to form conidia or sclerotia dependent on the illumination conditions, were subjected to the transcriptomic analysis, two sets of “developmental genes” among the light-responsive genes can be expected, those implicated in induction of conidiation or in suppression of sclerotial development.

**Figure 8:**
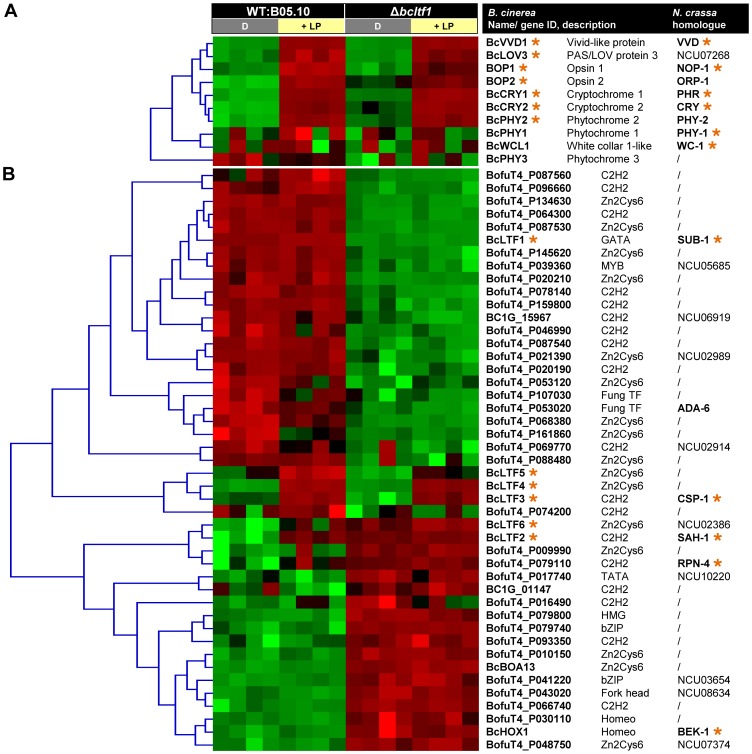
Differentially expressed genes may encode regulators of light-dependent differentiation. Relative expressions of the genes (i.e. log2-normalized intensities scaled by gene) in the four conditions (16 hybridizations) were clustered and depicted by color scale, where shades of green and red represent under- and overexpressed genes, respectively. Homologues in *N. crassa* were identified by a bidirectional best-hit (BDBH) approach. Light-induced genes in *B. cinerea* and *N. crassa* are marked with orange asterisks. For more details see Table S3. (**A**) Expression of most photoreceptor-encoding genes is similarly induced by light in wild type and Δ*bcltf1* strains. All predicted photoreceptors were clustered. (**B**) Expression levels of 45 TF-encoding genes are altered in response to light or deletion of *bcltf1*. TFs that were differentially expressed in at least one condition were subjected to clustering.

Light responses are based on the perception of light as a signal and the induction of subsequent signaling events. Though it is not clear why photoreceptors should be regulated on the level of gene expression, their light-responsive expression is a known phenomenon in *N. crassa* [37], [48]. Accordingly, expression of most of the predicted photoreceptor-encoding genes of *B. cinerea* was induced by light (Fig. 8A, Table S3). *Bcvvd1* encoding a putative blue light receptor was the strongest up-regulated gene with a 37-fold increase. Further light-induced genes are those encoding cryptochromes (*bccry1*, *bccry2*), opsins (*bop1*, *bop2*) and one of three phytochromes (*bcphy2*). Whereas similar intensities were found for *bcvvd1* in the wild type and the Δ*bcltf1* mutant, amplitudes of light induction of the other photoreceptor-encoding genes were much lower in the mutant than in the wild-type background as the genes in Δ*bcltf1* were already higher expressed in the dark (*bccry1*, *bccry2*, *bop2*, *bcphy2*) or less induced by light treatment (*bop1*, *bop2*) than in the wild type. *Bcwcl1* encoding a putative blue light-sensing transcription factor was not found among the light-induced genes in this microarray experiment. However, further analysis showed that *bcwcl1* is expressed higher during incubation in LL than in DD (data not shown), indicating that light also affects the expression of this gene. Among the light-induced genes *bclov3* (PAS/LOV protein 3) might be related to light perception as the deduced protein sequence shares similarities with N-terminal parts of plant phototropins. These proteins are autophosphorylating protein kinases harboring N-terminal blue light-sensory (LOV) and C-terminal kinase domains [49].

Regulators that function downstream of signaling cascades are transcription factors (TFs) whose activities might be transcriptionally and/or post-transcriptionally modified. The genome of *B. cinerea* contains 406 genes encoding TFs of different families [2], [50], 45 TF-encoding genes were differentially expressed in at least one of the four conditions, i.e. displaying a light response in wild type or Δ*bcltf1* mutant, or a Δ*bcltf1* effect in darkness or in response to light (Fig. 8B, Table S3). Hierarchical clustering of the selected TFs across the 16 hybridizations highlights that the differential expression is mainly due to the *bcltf1* mutation (43 TF-encoding genes) and to a smaller extent to the light condition (seven TF-encoding genes). In addition to *bcltf1*, the expression of five further TF-encoding genes was induced by light and the genes were accordingly named *bclft2*-*bclft6*. BcLTF2 and BcLTF3 are C2H2-TFs homologous to the light-responsive TFs SAH-1 and CSP-1 of *N. crassa* [37], [48]. BcLTF4 to BcLTF6 belong to the family of Zn2Cys6-TFs, and only a homologue of BcLTF6 is present in *N. crassa*. While the expression of *bcltf3*, *bcltf4* and *bcltf5* was similarly induced by light in both genomic backgrounds, the light-dependent expression pattern of the other two TF-encoding genes are affected in different ways in the absence of BcLTF1. Both *bcltf2* and *bcltf6* were overexpressed in the Δ*bcltf1* mutant in darkness, but only the expression level of *bcltf6* was further increased in response to the light stimulus. Therefore, BcLTF1 is involved in repressing the transcription of *bcltf2* and *bcltf6* in the dark. Moreover, it can be assumed that BcLTF1 represses the expression of 16 other TF-encoding genes in a light-independent manner, including *bcboa13* that belongs to the botcinic acid (BOA) gene cluster (see next section). The expression of one TF-encoding gene (BofuT4_P161860) was repressed by light in the wild type and the effect was found to be absent in the Δ*bcltf1* background, as the gene was no longer expressed (even in darkness). The gene encodes a member of the Zn2Cys6-TF family and is physically linked with genes of unknown functions displaying a similar expression pattern (*Bclft1g1499-1505*; Table S2).

### Absence of BcLTF1 affects expression of secondary metabolism-related genes

Fungal secondary metabolite (SM)-biosynthetic genes are typically located adjacent to each other and exhibit similar patterns of expression. These clusters usually include a gene encoding the key enzyme responsible for synthesis of the raw product, genes encoding enzymes for modifications and the transport of the compound, and genes whose products regulate cluster gene expression [51]. The genome of *B. cinerea* comprises 43 genes encoding SM key enzymes including 21 polyketide synthases (PKSs), nine non-ribosomal peptide synthetases (NRPSs), six sesquiterpene cyclases (STCs), three diterpene cyclases (DTCs) and one dimethylallyl tryptophan synthase (DMATS) [2]. In our transcriptomic approach, 38 of the 43 genes were significantly expressed in at least one condition (see Materials and Methods). The expression patterns of 17 of these genes (45%) are significantly altered in the absence of BcLTF1, including 9 PKS-, 3 NRPS-, 4 STC- and the single DMATS-encoding gene (Fig. S7A). Remarkably, the majority (15) of these BcLTF1-dependent genes are not responsive to light. This group includes *bcbot2* (*bcstc1*) that is part of the BOT-biosynthetic cluster (*bcbot1-5*) as well as *bcboa6* and *bcboa9* (PKSs) that belong to the two gene clusters (*bcboa1-6*, *bcboa7-17*) required for BOA biosynthesis. The other cluster genes were overexpressed as the key enzymes (Fig. S7B), suggesting that the mutants may produce more toxins under the tested conditions. Additionally, the two PKS-encoding genes *bcpks12* and *bcpks13* were under- and overexpressed in the Δ*bcltf1* mutant, respectively, and are considered to be associated with dihydroxynaphthalene (DHN)-melanin biosynthesis [41]. Beside the PKS yielding the polyketidic precursor, probably three other enzymes encoded by *bcbrn1*, *bcbrn2* and *bcsd1* are involved in melanin biosynthesis (Fig. S8). Increased expression levels of *bcpks13*, *bcbrn1*, *bcbrn2*, and *bcscd1* in Δ*bcltf1* mutants were associated with the dark pigmentation of the culture medium, while expression of *bcpks12* was exclusively detected in developing sclerotia of the wild type. Therefore, *bcpks13* and *bcpks12* are differentially expressed in Δ*bcltf1* mutants as well as during the life cycle of the wild type.

The expression patterns of only three key enzyme-encoding genes (*bcnrps2*, *bcphs1*, *bcstc5*) were significantly affected by light. Notably, one of them corresponds to a cluster of light-induced genes (*bcphs1*, *bcphd1*, *bccao1*) that encode homologues of enzymes involved in the biosynthesis of retinal, the chromophore for opsin, in *Fusarium fujikuroi* (phytoene synthase, phytoene dehydrogenase, and carotenoid oxygenase). As in *F. fujikuroi*, the cluster contains a forth co-regulated gene encoding an opsin (*bop2*) [52]–[54] (Fig. S9). Though genes were still induced by light in the absence of BcLTF1, the absolute expression levels were much lower than those found in the wild type in response to light, suggesting decreased retinal production and an altered function of BOP2 in the absence of BcLTF1.

### Light and BcLTF1 affect the expression of ROS-related genes

Given the finding that mutations of *bcltf1* affect the accumulation of H_2_O_2_, we inspected the transcriptomic data for genes involved in ROS metabolism to gain insight whether the net over-accumulation is due to an inappropriate detoxification and/or generation of ROS. Oxidative stress response (OSR) systems include superoxide dismutases (SODs) that convert O^−^_2_• into the less toxic H_2_O_2_, and catalases (CATs), peroxidases (PRDs), and peroxiredoxins (PRXs) that detoxify H_2_O_2_ and thereby prevent the formation of OH• via the Fenton reaction. Important non-enzymatic mechanisms include the oxidation of compounds such as glutathione, ascorbate and carotenoids. Reduced glutathione (GSH) provided by glutathione reductases (GLRs) is used by glutathione peroxidase (GPX), glutaredoxins (GRXs) as well as by glutathione S-transferases (GSTs) that conjugate GSH with various substrates such as peroxides [55]. In *B. cinerea*, several enzyme activities are represented by multiple genes, thus, four SODs, eight CATs, 16 PRDs, nine PRXs, one TRX, five GRXs and 26 GSTs have been identified (Table S4). The set of light-induced genes in the wild type is significantly enriched for OSR-related genes (Table 2), among them are two CATs (*bccat3, bccat4*), two PRDs (*bcprd1, bcprd2*) and three GSTs (*bcgst3, bcgst8, bcgst15*) (Table S4). These genes are not induced by light in the absence of *bcltf1* suggesting that their products may contribute to coping with light-related oxidative stress. Nevertheless, twenty genes among them SOD-, CAT-, PRD-, PRX- and GST-encoding genes exhibited increased or decreased expression levels in the mutant background, reflecting a general deregulation of OSR-related genes.

Several enzymes of *B. cinerea* may generate ROS either as secondary messenger (e.g. formation of O^−^_2_• by the NADPH oxidase (NOX)) or as side products of metabolic processes (e.g. oxidation of carbohydrates, fatty acids and amino acids yielding H_2_O_2_). Forty-eight putative oxidase-encoding genes were identified in the genome, and expression levels of twenty-five genes were affected by either the light stimulus and/or the deletion of *bcltf1*. Four oxidase-encoding genes were induced by light in both genomic backgrounds, and a further 11 oxidase-encoding genes exhibited increased expression levels in the mutant background. Seven out of the 11 identified DAO (D-amino acid oxidase)-encoding genes are less expressed in the *bclft1* mutant than in the wild type, irrespective of the illumination condition, a fact that might be related with the weak expression of amino acid transporter-encoding genes (Table 2). Additionally, two out of the four identified putative NADH:flavin oxidoreductase/NADH oxidase-encoding genes showed increased expression levels in the absence of BcLTF1 while the genes encoding the catalytic subunits of the NOX complex (*bcnoxA* and *bcnoxB*) were slightly underexpressed (Table S4).

### Absence of BcLTF1 results in increased alternative respiration

Beside the mentioned ROS-generating enzymes, the mitochondrial electron transport chain (ETC) massively contributes to intracellular ROS levels. Complexes I and III are forming O_2_^−^ that is in turn dismutated to H_2_O_2_ to prevent the formation of OH• [56]. Though expression levels of nuclear-encoded subunits of the different protein complexes of the respiratory chain were not affected in the Δ*bctlf1* mutant (data not shown), strongly increased expression levels were observed for the gene encoding the alternative oxidase (BcAOX1) (Table S4, Fig. S10). This enzyme accepts electrons from the ubiquinone pool and reduces oxygen directly, hence bypassing the electron flux through complexes III and IV (cytochrome c oxidase, COX), and related ROS generation [57]. In addition to complex I (NADH:ubiquinone oxidoreductase), *B. cinerea* possesses five putative NADH dehydrogenases that may deliver electrons to the ubiquinone pool thereby bypassing complex I. In fact, *bcnde2* and *bcndi1* encoding external and internal NADH dehydrogenases, respectively, were overexpressed in the deletion mutants. Additionally, genes encoding cytochrome c (*bccyc1*), a cytochrome c peroxidase (*bcccp2*), an uncoupling protein (*bcucp1*) and several other mitochondrial carrier proteins were more highly expressed in the absence of BcLTF1, further supporting the deregulation of the ETC in the mutant background. CCP2 may accept electrons from complex III/cytochrome c for reduction of H_2_O_2_, hence avoiding the electron flux through COX [58], and uncoupling proteins are considered to decrease ROS production by facilitating the transport of ions across the membrane [56], [59]. Taken together, the observed transcriptional changes in the Δ*bcltf1* mutant suggest that alternative enzymes compete with the ETC complexes for electrons to decrease the generation of mitochondrial ROS.

## Discussion

Random mutagenesis via ATMT is successfully used in plants and filamentous fungi to generate new phenotypes and to identify the corresponding genes. In a recent study, we reported on an ATMT approach in *B. cinerea* yielding 2,367 mutants with 560 exhibiting reduced virulence in a first screening. For two out of four randomly chosen candidates tested, the genes could be linked to the ATMT phenotype by deletion approaches. As reported for other fungal ATMT libraries, T-DNA integrations were mostly random, single copy, and occurred preferentially in noncoding (regulatory) regions [39]. The latter facts hold true for the ATMT mutants we are describing in this study, however, the fact that three independent mutants out of 77 virulence-attenuated mutants analyzed so far were found to contain T-DNA insertions at the same gene locus, indicates that “hot spots” for T-DNA insertions exist in the genome of *B. cinerea*.

While the deletion of *bcltf1* mainly affects the advanced stages of infection, the overexpression of *bcltf1* impairs specifically the penetration process by germ tubes which exhibit an abnormal branching pattern. In contrast, penetration via infection cushions from already established mycelia is not impaired indicating that these modes of penetration are independently regulated processes. This is in agreement with previous reports on mutants that are impaired in mycelia- but not in conidia-derived infection [60]. The retarded colonization of the host tissue by Δ*bcltf1* mutants is accompanied and likely caused by increased accumulation of ROS as the addition of antioxidants to the inoculation droplets restored virulence to wild-type levels. Since the deletion mutants accumulate more H_2_O_2_ than the wild type under axenic conditions, the increased H_2_O_2_ levels in Δ*bcltf1*-infected plant tissues probably are the consequence of inappropriate ROS production by the fungus rather than by the plant. However, the increased accumulation has a negative impact on invasion of the host tissue by the mutant, whereas the application of antioxidants does not interfere with virulence of the wild type, raising the question to which extent ROS produced by the host (in terms of an oxidative burst) or the fungus are contributing to the outcome of the interaction.

Homologues of BcLTF1 have been characterized so far only in very few fungal species, revealing their requirement for sexual reproduction in Sordariomycetes (*N. crassa, S. macrospora*), and Eurotiomycetes (*A. nidulans* and *A. fumigatus*) [33], [35], [36], [38]. Due to the lack of sclerotia, Δ*bcltf1* as well as OE::*bcltf1* mutants are female sterile, and for crossings of Δ*bcltf1* microconidia (*MAT1-1*) with sclerotia of the reference strain SAS405 (*MAT1-2*) no apothecia were obtained (data not shown) indicating that the TF is required for sexual reproduction in *B. cinerea* as well. However, significant differences also exist that may reflect the requirement to adapt to different ecological niches. NsdD in *A. flavus* clearly regulates the mode of asexual reproduction, either via formation of conidia or sclerotia in a light-independent manner [61].

Regardless of the fact that the mentioned fungi are distantly related and exhibit very different modes of reproduction including different asexual and sexual structures, reproduction is controlled by different environmental cues. Therefore, protoperithecia formation in *N. crassa* requires nutrient limitation and light [19], and limited air exchange favors sexual reproduction in *A. nidulans* while nutrient limitation, light and other stresses shift the ratio from sexual to asexual reproduction [25]–[26]. On the contrary, light alone controls asexual reproduction in *B. cinerea* by inducing conidiation and suppressing sclerotial development preventing thereby the simultaneous formation of both structures. In contrast, the formation of microconidia is induced by nutrient limitation [62], and also the formation of apothecia in the laboratory takes place in the absence of nutrients and requires a photoperiod [10]. Hence, light has an outstanding function in *B. cinerea* because it determines the mode of reproduction.

In accordance with the findings that *B. cinerea* undergoes photomorphogenesis and that its germ tubes, conidiophores and apothecia stipes exhibit phototropic responses [63], a number of light-responsive genes (293) could be identified by genome-wide expression analysis. Similar studies have revealed 314, 533, and 250 light-responsive genes in *N. crassa*, *A. nidulans*, and *A. fumigatus*, respectively [37], [64]–[65]. Fifteen min of white light was sufficient to induce the transcription of *bcltf1* and two other tested light-responsive genes (*bop1*, *bcccg1*), and maximal induction was achieved at exposure times of 45–60 min. BcLTF1 is an important modulator of the transcriptional responses to light as it influences the expression patterns of the majority of light-responsive genes (66%). Similar observations have been made for SUB-1 in *N. crassa* which acts in a hierarchical light-sensing cascade, being – as an early light-responsive gene – involved in induction of the majority of late light-responsive genes [37]. Noteworthy, transcript levels of both *bcltf1* and *sub-1* increase in response to light but no light responsiveness of *A. nidulans nsdD* has been reported [64], indicating that NsdD is unlikely to be involved in light signaling in the same fashion as its homologues in *B. cinerea* and *N. crassa*.

White light is composed of different wavelengths; therefore different photoreceptors are assumed to be involved in perception and subsequent signaling. The inducible effect of the blue light fraction on expression of *bcltf1* is likely mediated via a WHITE COLLAR-like transcriptional complex (WCC) formed by the GATA transcription factors BcWCL1 and BcWCL2 that were reported to interact in the nuclei [66]. In fact, induction of *bcltf1*, *bop1* and *bcccg1* by white light requires BcWCL1 because in its absence the expression levels are much lower than in the wild type [P. Canessa, J. Schumacher, M. Hevia, P. Tudzynski, L. Larrondo, unpublished]. Nevertheless, an induction is still visible suggesting the involvement of other photoreceptors/wavelengths in transcriptional activation of *bcltf1* and other light-induced genes. Δ*bcltf1* mutants exhibit similarly decreased radial growth rates in blue and white light suggesting that the blue light fraction accounts for the toxicity of white light, possibly by provoking greater oxidative stress than other wavelengths. Remarkably, the light-protecting function seems to be unique to BcLTF1 because *N. crassa* Δ*sub-1* and *S. macrospora* Δ*pro44* mutants exhibit comparable vegetative growth in light and darkness [L. Larrondo, pers. commun., 38].

The observation that expression levels of light-induced genes (*bop1*, *bcccg1*) decreased after prolonged exposure to light (from 120 min on) indicates the existence of photoadaptation processes in *B. cinerea*. In *N. crassa*, photoadaptation involves a further blue light-sensing protein (VIVID), whose expression is induced by light in a WCC-dependent manner, and that then negatively acts on the WCC to prevent further activation of light-responsive genes [67]–[68]. The homologue of *B. cinerea* (*bcvvd1*) is highly induced by light in both wild-type and Δ*bcltf1* strains, however, whether BcVVD1 impacts light-induced gene expression and is required for light tolerance like its counterpart in *N. crassa* needs to be studied. VIVID, the WCC and the FREQUENCY (FRQ) protein are furthermore implicated in running the circadian clock in *N. crassa*. Like *vvd*, expression of *frq* is turned on by the WCC in response to light, and subsequently FRQ blocks its own transcription by physically interacting with the WCC [20]. *B. cinerea* possesses a homologue of *frq* whose expression is induced by light in wild type and the *bcltf1* deletion mutants to a similar extent, suggesting that the circadian clock is set by light in a BcLTF1-independent manner, as it was described for SUB-1 in *N. crassa* [37]. The fact that expression of *bcltf1* in contrast to its homologue in *N. crassa* is not subjected to photoadaptation underlines the importance of the transcription factor for mediating the tolerance towards light in *B. cinerea*.

Based on the finding that the deletion of BcLTF1 results in hyperconidiation, it can be concluded that BcLTF1 acts as a general repressor of conidiation. This result was unexpected because the light-responsiveness of *bcltf1* may suggest a stimulatory role of the TF on light-dependent differentiation processes. Additionally, a direct role for BcLTF1 in the induction of sclerotia development appears unlikely as its overexpression results in the accumulation of undifferentiated aerial mycelia instead of sclerotia. This fluffy phenotype resembles those of wild type colonies that have been illuminated with blue light [15]–[16] supporting the role of BcLTF1 in mediating blue light responses. Hence, it is concluded that conidiation is initiated by the differentiation of aerial hyphae (triggered by blue light, involving WCC and BcLTF1) followed by their differentiation to conidiophores (possibly triggered by the other light wavelengths/photoreceptors).

In addition to BcLTF1 that represses conidiation in light and darkness, five other light-responsive TFs were identified that may regulate differentiation in a light-dependent fashion. Homologues of *bcltf2* and *bcltf3* in *N. crassa* (*sah-1*/*short aerial hyphae* and *csp-1*/*conidial separation*) are inducible by light and control asexual development [36]–[37]. Increased expression levels of *bcltf2* in the Δ*bcltf1* background in darkness correlate with the hyper-conidiation phenotype of the mutant, suggesting that BcLTF2 indeed may act as a positive regulator of conidiation. Light-responsive TFs may be also implicated in suppressing sclerotial development in the light, for instance by induction of transcriptional repressor(s) of sclerotia-related gene expression. Further studies on the light-responsive TFs are envisaged to reveal whether and how they are involved in differentiation of reproductive structures. Likewise these TFs may be involved in regulation of DHN-melanin biosynthesis, a pigment that is incorporated in the reproductive structures giving conidiophores, conidia and sclerotia of *B. cinerea* their characteristic gray to black color [69]–[70]. Accordingly, Δ*bcltf1* and Δ*bcvel1* mutants [31]–[32] exhibiting a hyper-conidiation phenotype produce melanin in excess leading to a dark coloration of the culture broths. In contrast to other DHN-melanin-producing fungi, *B. cinerea* possesses two highly similar PKS-encoding genes (*bcpks12* and *bcpks13*) [41], while single copies for the other melanogenic genes exist. The relevance of having two copies of the PKS appears obscure when both enzymes are functional. However, considering the differential expression pattern of *bcpks12* and *bcpks13* in the Δ*bcltf1* background and during the life cycle of the wild type and the fact that *B. cinerea* produces, in contrast to other fungi, the same pigment under very different conditions, it is conceivable that BcPKS12 and BcPKS13 are responsible for sclerotia- and conidiophore/conidia-specific melanin biosynthesis, respectively. The observation that non-sporulating *B. cinerea* mutants produce an orange pigment during intensive illumination suggests furthermore that the fungus is able to produce carotenoids, a group of pigments that is produced by plants and fungi to protect cells from free radicals, ROS and singlet oxygen [71]. Consequently, increased carotenoid production may result in extended life spans as demonstrated in *Podospora anserina* [72]. Genes putatively involved in retinal biosynthesis in *B. cinerea* are inducible by light as their homologues in *N. crassa* and *F. fujikuroi*, but unlike the situation in *N. crassa*, genes in the two other fungi are organized together with the opsin-encoding gene in a gene cluster [37], [54]. Nevertheless, as described in *N. crassa*, light-induction of carotenogenesis in *B. cinerea* relies on the WCC [P. Canessa, J. Schumacher, M. Hevia, P. Tudzynski, L. Larrondo, unpublished]. Given the protective function of the carotenoids, their decreased production by Δ*bcltf1* mutants, as concluded from lower gene expression levels, may contribute to the hypersensitivity of the mutant to light and oxidative stress.

Several observations suggest that BcLTF1 is essential for maintaining the ROS homoeostasis. Expression levels of many ROS-related genes are altered in the absence of BcLTF1, though it is still ambiguous which of the changes (decreased scavenging, increased generation in the peroxisomes and/or mitochondria) cause the significantly increased net accumulation of H_2_O_2_ and therefore rendering Δ*bcltf1* mutants more sensitive to oxidative stress arising from exogenous ROS, light, and during infection. Some but not all phenotypes of Δ*bcltf1* mutants can be restored by antioxidants illustrating that the unbalanced ROS homoeostasis accounts for decreased virulence and mediates light toxicity, but it does not cause the observed developmental defects. Although clear evidences suggest an altered cellular redox status in the deletion mutants, the use of roGFP2 as sensor failed to confirm that, because the initial cytosolic glutathione pool is reduced and recovers after a short-term oxidation event caused by H_2_O_2_ in a manner almost as in the wild-type. However, given that the equilibrium of the glutathione pool is limited between different cellular compartments, as it was reported for plant cellular compartments [73], the mutant may experience oxidative stress in another compartment other than the cytosol. The overexpression of nuclear genes encoding enzymes of the alternative respiration pathway in Δ*bcltf1* mutants indicates the reprogramming of the mitochondria probably as a consequence of their dysfunction (retrograde signaling) [74]. The key component of the alternative pathway is the AOX that functions as the terminal oxidase (instead of the copper-containing COX in cytochrome ETC) transferring electrons to oxygen. Studies in different fungi reported an increased *aox* expression level in response to the inhibition of later steps of cytochrome ETC [75]–[78], to oxidative stress [79]–[82], and to copper deficiency causing COX assembly defects [83]–[84]. AOX activity has been reported to be dispensable for virulence in *M. oryzae*, *B. cinerea* and *A. fumigatus*, however, in the latter it contributes to oxidative stress resistance [85]–[86], [77; C. Levis, pers. commun.]. In *P. anserina*, AOX activity in complex III-deficient mutants leads to an extended lifespan associated with a decreased ROS production [87], [76]. On the contrary, the long-lived phenotype of mutants with a nonfunctional COX (deletion of the copper chaperone PaCOX17) is accompanied by increased ROS production [88]. Accordingly, studies in plant cells demonstrated the oxidation of the mitochondrial glutathione pool in response to the inhibition of COX (without affecting the cytosolic redox status), but not in response to inhibition of complex III [89]. From the present data it is difficult to define the reason for increased alternative respiration in Δ*bcltf1* mutants, i.e. whether increased overall ROS production or an impairment of the cytochrome ETC causes the expression of the corresponding genes. However, the observation that the homologues of the copper chaperone COX17 and the high-affinity copper transporter CTR3 are significantly overexpressed in the Δ*bcltf1* mutant may indicate an altered copper homoeostasis in the mutants which in turn may affect the activity of COX and other enzymes requiring copper as cofactor.

Regardless of how increased alternative respiration is achieved, the altered mode of respiration is expected to reduce mitochondrial ROS production and to cause further global changes in the metabolism of the fungus, because the electron flux through the alternative enzymes is not coupled with the translocation of protons across the membrane and therefore does not allow for proton gradient-driven ATP synthesis. Retrograde signaling in yeast cells was shown to affect glycolysis, the tricarboxylic acid cycle, peroxisomal fatty acid oxidation as well as the glyoxylate cycle [90]. We suggest therefore that the significantly altered secondary metabolism in Δ*bcltf1* mutants is due to an altered primary metabolism rather than to a direct involvement of BcLTF1 in regulation of secondary metabolism-related genes.

**Figure 9:**
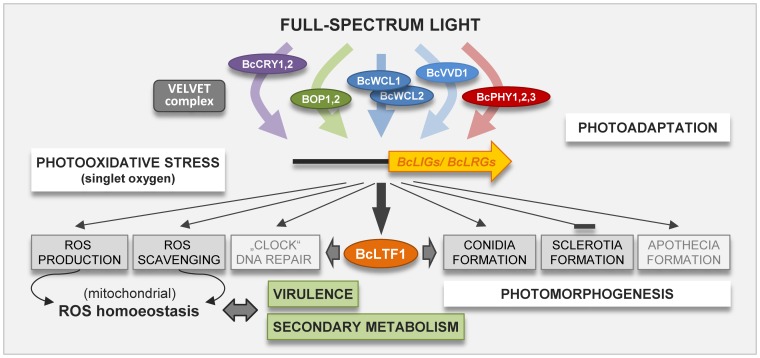
Photoresponses in *B. cinerea* are modulated by the light-responsive transcription factor BcLTF1. Light determines the mode of asexual reproduction (conidia vs. sclerotia), is required for differentiation of the fruiting bodies (apothecia) and is further considered to set the circadian clock. The cellular redox status is affected by emerging singlet oxygen and other ROS; compensation is achieved by induction of antioxidant systems including enzymes and carotenoids. *Bcltf1* represents one of the 244 light-induced genes, and its absence affects several light-dependent features such as the formation of reproductive structures. The deletion of BcLTF1 leads to an unbalanced ROS status (net overproduction of H_2_O_2_), possibly in the mitochondria, as suggested by the upregulation of the alternative respiration pathway. The altered ROS status is considered to cause the inability of the deletion mutant to tolerate additional oxidative stress that arises during illumination (singlet oxygen), exposure to ROS (H_2_O_2_, menadione) and during host infection (oxidative burst).

Taken together, it can be assumed that light affects *B. cinerea* in two ways (Fig. 9); directly via generation of singlet oxygen that damage macromolecules, and indirectly via its sensing by fungal photoreceptors altering gene expression. Products of light-responsive genes are implicated in ROS homoeostasis (via generation or scavenging), photoadaptation, the circadian clock, in promoting conidiation and apothecia formation and in preventing sclerotial development. Many, but not all, responses are altered in the absence of BcLTF1; and a hallmark of the deletion mutants is the unbalanced ROS homoeostasis (net ROS overproduction) accompanied by a retrograde response (alternative respiration) accounting for reduced virulence, light toxicity and potentially for altered secondary metabolism. Phenotypic and expression data suggest that BcLTF1 exerts repressing as well as activating functions in constant darkness and in response to light, though its transcription level is very low in darkness.

## Materials and Methods

### *B. cinerea* strains and growth conditions

Strain B05.10 of *B. cinerea* Pers. Fr. [*B. fuckeliana* (de Bary) Whetzel] is an isolate from *Vitis* (Table 1) and is used as the recipient strain for genetic modifications. Genome sequences of B05.10 and T4 were recently published [2], [91]. Strains were cultivated on plates containing solid synthetic complete medium (CM) [92], modified Czapek-Dox (CD) as minimal medium (2% sucrose, 0.1% KH_2_PO_4_, 0.3% NaNO_3_, 0.05% KCl, 0.05% MgSO_4_×7 H_2_O, 0.002% FeSO_4_×7 H_2_O, pH 5.0), or solid potato dextrose medium (Sigma-Aldrich, Germany) supplemented with 10% homogenized bean leaves. Acidification of the culture medium (solid CM pH 8) was monitored using the pH indicator bromothymol blue (0.1%) (Sigma-Aldrich, Germany). Cultures were incubated at 20°C under white light (12 h light/12 h darkness (LD) or continuous light (LL)) for conidiation, and in continuous darkness for induction of sclerotial formation. White light (9000 lx at culture level) was generated by using a set of Sylvania Standard F18W/29-530 “warm white” and F36W/33-640 “cool white” fluorescent bulbs. Wavelengths of transmitted light were controlled using Petri dish chambers covered with Roscolux polyester filters (#381 Baldassari Blue, #389 Chroma Green, #312 Canary, #27 Med Red; http://www.rosco.com/filters/roscolux.cfm); transmission spectra of the filters are shown in Fig. S3. Crossings using SAS405 (*MAT1-2*) as reference strain were performed as described previously [31]. For DNA and RNA isolation, mycelia were grown on solid CM medium with cellophane overlays.

### Standard molecular methods

Fungal genomic DNA was prepared according to Cenis [93]. For Southern blot analysis, fungal genomic DNA was digested with restriction enzymes (Fermentas, Germany), separated on 1% (w/v) agarose gels and transferred to Amersham Hybond-N+ filters (GE Healthcare Limited, UK). Blot hybridizations with random-primed α-^32^P-dCTP-labelled probes were performed as described previously [94]. PCR reactions were performed using the high-fidelity DNA polymerase Phusion (Finnzymes, Finland) for cloning purposes and the BioTherm Taq DNA Polymerase (GeneCraft, Germany) for diagnostic applications. Replacement fragments and expression vectors were assembled in *Saccharomyces cerevisiae* by exploiting its homologous recombination machinery [36], [66]. Sequencing of DNA fragments was performed with the Big Dye Terminator v3.1 sequencing kit (Applied Biosystems, USA) in an ABI Prism capillary sequencer (model 3730; Applied Biosystems). For sequence analysis, the program package DNAStar (Madison, USA) was used. Protocols for protoplast formation and transformation of *B. cinerea* were described by Schumacher [66]. Regenerated protoplasts were overlaid with SH agar containing 70 µg/ml hygromycin B (Invitrogen, The Netherlands) and 140 µg/ml nourseothricin (Werner-Bioagents, Germany), respectively. Resistant colonies were transferred to agar plates containing CM medium supplemented with the respective selection agent in a concentration of 70 µg/ml. Single conidial isolates were obtained by spreading conidial suspensions on selective media and transferring single colonies to new plates.

### Northern blot analyses

Total RNA was isolated making use of the TRIzol reagent (Invitrogen, The Netherlands). Samples (25 µg) of total RNA were transferred to Hybond-N+ membranes after electrophoresis on a 1% (w/v) agarose gel containing formaldehyde, according to the method of Sambrook et al. [95]. Blot hybridizations were carried out by use of random-primed α-^32^P-dCTP-labelled probes [94]. Analyzed genes are (Fig. 2): *Bcssp1* (BC1G_03185) encoding the homologue of *S. sclerotiorum ssp1* [40]; *bcpks12* (AY495617) and *bcpks13* (AY495618) encoding polyketide synthases putatively involved in melanin biosynthesis [41]; *bop1* (BC1G_02456) encoding an opsin-like protein, expression is induced upon H_2_O_2_ [30] and light treatment (this study); *bcccg1* (BC1G_11685) representing the homologue of *N. crassa ccg-1* [42]), expression is induced upon H_2_O_2_ and light treatment (this study); *bcactA* (AJ000335) encoding actin is used in this study to detect fungal RNA in mixed (*in planta*) samples; *bcgpx1* (BC1G_02031) encoding the glutathione peroxidase, expression is induced upon H_2_O_2_ treatment [96].

### Identification of *bcltf1* as virulence-associated gene

Strains PA31, PA2411 and PA3417 belong to an insertional mutant library of *B. cinerea* B05.10 that was generated by *A. tumefaciens*-mediated transformation (ATMT). Southern blot analyses demonstrating the existence of single T-DNA copies in the three mutants (data not shown) were performed according to Giesbert et al. [39]. The T-DNA-flanking regions were identified by TAIL-(thermal asymmetric interlaced) PCR as described previously [39]. BlastN analyses using the amplified T-DNA flanking regions revealed discontinuous sequences at the respective locus in both genome sequences of T4 (1-kb gap upstream of BofuT4_P09344; 480 aa) and B05.10 (2-kb gap upstream of BC1G_10441; 133 aa) (*B. cinerea* genome project, URGI; http://urgi.versailles.inra.fr/Species/Botrytis/) [2]. Re-sequencing of both strains yielded continuous sequences (*B. cinerea* Database, Broad Institute; http://www.broadinstitute.org/annotation/genome/botrytis_cinerea/ [91], hence, the T-DNA insertion occurred 1.608 kb upstream of the ORF hereafter called *bcltf1* (Fig. 1B). *Bcltf1* of B05.10 encodes a protein of 481 aa (BcLTF1^B05.10^: B0510_3555), that of T4 a protein of 480 aa (BcLTF1^T4^: BofuT4_P09344, BcT4_9306). Putative transcriptional start (TSS) and PolyA signals (Fig. 1B) were predicted using the FGENESH tool (http://linux1.softberry.com/). The nuclear localization signal (NLS) and the GATA zinc finger motif were predicted by WoLF PSORT (http://wolfpsort.org/) and Pfam Search (http://pfam.sanger.ac.uk), respectively (Fig. 1C). No nuclear export signals (NES) were identified by using NetNES 1.1 (http://www.cbs.dtu.dk/services/NetNES/). Putative phosphorylation sites for protein kinase A (PKA: S^78^, S^95^, S^184^, S^185^) and protein kinase C (PKC: T^91^, S^105^, S^124^, S^194^, T^453^, T^457^, S^467^, S^468^) in BcLTF1^B05.10^ were identified by running NetPhosK (http://www.cbs.dtu.dk/services/NetPhosK/).

### Construction of *bcltf1* mutants

For generation of fragments mediating the replacement of *bcltf1* (Δ*bcltf1*-A) and *bcltf1*+1.7 kb of the 5′-noncoding region (Δ*bcltf1*-B), respectively, different 5′ flanks (A, B) were assembled with the same 3′ flank and a hygromycin resistance cassette by yeast recombinational cloning [36] (Table S1, Fig. S1A). Replacement fragments were transformed into *B. cinerea* B05.10. Homologous integration events in hygromycin-resistant transformants were detected by diagnostic PCR using the primers pCSN44-trpC-T and pCSN44-trpC-P, binding in the hygromycin resistance cassette and the primers *bcltf1*-A-hi5F/B-hi5F and *bcltf1*-hi3R, binding upstream and downstream of the *bcltf1*-flanking regions, respectively (Table S1, Fig. S1B). Single spore isolates were screened for the absence of *bcltf1* alleles using primers *bcltf1*-WT-F and *bcltf1*-WT-R matching the substituted coding region. For Southern blot analysis, genomic DNA of the mutants and the recipient strain B05.10 was digested with *Pst*I, and the blot was hybridized with the 3′ flank of the replacement fragment. The hygromycin resistance cassette contains an additional *Pst*I restriction site resulting in smaller hybridizing fragments in the replacement mutants (2.3 kb) than in the wild type (7.2 kb) (Fig. S1C). Strain Δ*bcltf1*-A1 was discarded because a second ectopic integration event occurred. Taken together, two independent mutants were generated lacking either only *bcltf1* (Δ*bcltf1*-A6) or *bcltf1* and its 5′-noncoding region (Δ*bcltf1*-B1). The mutant Δ*bcltf1*-A6 was complemented by targeted integration of *bcltf1* or *bcltf1-gfp* at the native gene locus thereby replacing the hygromycin resistance cassette. For vector construction, all fragments indicated in Fig. S1A were amplified by PCR using oligonucleotides listed in Table S1, and assembled in one step in *S. cerevisiae* yielding plasmids p*bcltf1*-COM and p*bcltf1-gfp* ^in loco^. Replacement constructs were transformed into Δ*bcltf1*-A6, and diagnostic PCR confirmed the targeted integration in two transformants per construct (*bcltf1* ^COM^:T2, T3; and Δ*bcltf1*+P*bcltf1*::*bcltf1-gfp*: T2, T5) (Fig. S1D). For over-expressing the BcLTF1-GFP fusion protein, the ORF was introduced in vector pNAN-OGG comprising gene flanks for targeted integration at *bcniiA* (locus of nitrite reductase), a nourseothricin resistance cassette and the expression cassette with *gfp* under control of the constitutive *oliC* promoter [66] (Fig. S1E). The construct was transformed into B05.10 yielding transformants WT+P*oliC*::*bcltf1-gfp* (T6, T7), and into Δ*bcltf1*-B1 yielding transformants Δ*bcltf1*+P*oliC*::*bcltf1-gfp* (T3, T4). Targeted integration at *bcniiA* was verified by diagnostic PCR as described previously [66] (data not shown). Northern blot analyses revealed the constitutive expression of *bcltf1-gfp* in the mutant background and the overexpression of *bcltf1-(gfp)* in the wild-type background, respectively (Fig. S1F).

### Germination and germling fusion assays

Conidial germination was monitored according to Doehlemann et al. [97]. For testing nutrient- and hydrophobicity-induced germination, cleaned conidia were incubated in Gamborg B5+10 mM glucose on glass surfaces and in double distilled water on polypropylene foil, respectively, in LL or DD conditions. Germination rates were determined in triplicates after 4 h and 8 h of incubation for glucose-induced and after 24 h for polypropylene-induced germination. Germling fusions via conidial anastomosis tubes were monitored according to Roca et al. [98]. Briefly, conidia were plated on solid Vogel's medium and incubated for 17 h at 21°C in the dark. Samples were analyzed by using light microcopy.

### Virulence assays

For penetration assays, onion epidermal layers were prepared as described [31]. Conidial suspensions or non-sporulating mycelia from 3-d-old cultures were used for inoculation. The staining with lactophenol aniline blue (Sigma-Aldrich, Germany) allowed the identification of the penetrations sites after 24 h of incubation. For infection assays on French bean (*Phaseolus vulgaris* cv. 90598), primary leaves of living plants were inoculated with 7.5-µl droplets of conidial suspensions (2×10^5^ conidia/ml Gamborg B5+2% glucose) or non-sporulating mycelia [31]. Infected plants were incubated in plastic boxes at 22°C under natural illumination conditions (LD) and continuous darkness (DD), respectively. Trypan blue and DAB (3,3′-diaminobenzidine) staining of lesions were performed as described previously [31]. Bright field images were taken using the Zeiss SteREO Discovery V.20 stereomicroscope or the AxioScope.A1 microscope equipped with an AxioCamMRc camera and the AxiovisionRel 4.8 software package (Zeiss, Germany).

### Detection of reactive oxygen species (ROS)

To detect accumulating H_2_O_2_, strains were grown on solid CM covered with cellophane for 3 d in DD. Fresh mycelia (25 mg) were weighed, placed in wells of a 24-well plate and flooded with 1 ml DAB solution (0.5 mg/ml DAB in 100 mM of citric acid buffer, pH 3.7). Samples were incubated for 2 h in darkness at room temperature. The colorless DAB is oxidized by H_2_O_2_ in the presence of fungal peroxidases and horseradish peroxidase (positive control) resulting in a brown coloration of the staining solution. To detect accumulating O_2_.^−^, germinated conidia were incubated for 2 h in NBT staining solution (0.5 mg/ml nitrotetrazolium blue chloride in 50 mM sodium phosphate buffer, pH 7.5). Samples were analyzed by using light microcopy. The colorless substrate NBT is converted to a blue precipitate in the presence of O_2_^−^.

### Measurement of the cellular glutathione redox potential

Vector pNAN-GRX1-roGFP2 carrying the reduction-oxidation sensitive green fluorescent protein (roGFP) and a nourseothricin resistance cassette [45], was transformed into the mutant Δ*bcltf1*-A6 (Table 1). The homologous integration of the construct at *bcniiA* in two independent transformants (T2, T3) was verified by diagnostic PCR as described previously [66]. Reactions of roGFP2 in wild type B05.10 (WT+*grx-rogfp2*) and *bcltf1* deletion mutants (Δ*bcltf1*+*grx-rogfp2*) were monitored using a fluorometer as described by Heller et al. [45]. Briefly, germinated conidia (conidia cultivated for 12 h in liquid CM) were washed twice and transferred to 96-well plates. Fluorescence was measured at the bottom with 3×3 reads per well and an excitation wavelength of 395±5 nm for the oxidized state and 488±5 nm for the reduced state of roGFP2 using a Tecan Safire fluorometer. Relative fluorescence units (RFU) were recorded to calculate the Em_395_/Em_488_ ratio.

### Fluorescence microscopy

For microscopy, conidia were suspended in Gamborg B5 solution supplemented with 2% glucose and 0.02% ammonium phosphate, and incubated overnight under humid conditions on microscope slides or on onion epidermal strips. Nuclei were stained using the fluorescent dye Hoechst 33342 [66], and fungal cell walls using calcofluor white (CFW) as described previously [60]. Fluorescence and light microscopy was performed with a Zeiss AxioImager.M1 microscope equipped with the ApoTome.2 technology for optical sectioning with structured illumination. Differential interference microscopy (DIC) was used for bright field images. Specimens stained by Hoechst 33342 and CFW were examined using the filter set 49 DAPI shift free (excitation G 365, beam splitter FT 395, emission BP 445/50), GFP fluorescence using filter set 38 (excitation BP 470/40, beam splitter FT 495, emission BP 525/50). Images (optical sections and Z-stacks) were captured with an AxioCam MRm camera and analyzed using the Axiovision Rel 4.8 software package (Zeiss, Germany).

### Microarrays analyses

Wild type B05.10 and the Δ*bcltf1* mutant were grown for 3 d on solid CM in LD before non-sporulating mycelia were transferred to solid CM covered with cellophane overlays. Cultures were incubated for 52 h in DD, and then half of the cultures were transferred to white light (+LP). After 60 min all cultures (WT-D; WT+LP; Δ*bcltf1*-D; Δ*bcltf1*+LP) were harvested. Material derived from four independent experiments was used for RNA isolation (Trizol procedure). Total RNA was treated with the DNA-free kit to remove any trace of DNA (Ambion - Applied Biosystems, France). Synthesis of double-stranded cDNA, Cy3-labeling and hybridization on microarrays were done by PartnerChip (http://www.partnerchip.fr/) using the procedures established by NimbleGen (Roche) and the reagents from Invitrogen (Life technologies, France). To study the complete transcriptome of *B. cinerea*, NimbleGen 4-plex arrays with 62,478 60-mer specific probes covering all the 20,885 predicted gene models and non-mapping ESTs of *B. cinerea* [2] were used. The arrays also include 9,559 random probes as negative controls. Data processing, quality controls and differential expression analysis were performed using ANAIS methods [99]. Probe hybridization signals were subjected to RMA-background correction, quantile normalization, and gene summarization [100]–[101]. Genes with a normalized intensity over the threshold (1.5×95^th^ percentile of random probes) in at least one hybridization were considered as expressed and kept for further analyses. Differentially expressed genes (light response in wild type and mutant, Δ*bcltf1* effect in darkness and light) were then identified using a one-way ANOVA test. To deal with multiple testings, the ANOVA p-values were then submitted to a False Discovery Rate correction. Genes with a corrected p-value <0.05, and more than a 2-fold change in transcript level were considered as significantly differentially expressed. BlastN analyses for the 20,885 spotted genes [2; URGI database] were performed to identify the matching gene models derived from the re-sequencing approaches by Staats & van Kan [91; Broad Institute]. By this, the number of differentially expressed genes was reduced because incorrect gene models derived from discontinuous sequences were revised and furthermore previously non-matching ESTs could be mapped. The four gene lists were then pooled and cluster analyses were performed to highlight the different expression profiles encountered. For this purpose, the log2-normalized intensities scaled by gene across the 16 hybridizations were clustered using Genesis tools [102]. Gene enrichment analyses were further performed with GSEA toolkit [46]–[47] to highlight significantly enriched functions compared to the complete list of functionally annotated *B. cinerea* genes [2]. Details on the experiments, raw and normalized values are available at NCBI GEO (http://www.ncbi.nlm.nih.gov/geo/) (Accession: GPL17773). Furthermore, lists of differentially expressed genes are available at http://urgi.versailles.inra.fr/Data/Transcriptome.

## Supporting Information

Figure S1 Construction of *bcltf1* mutants. (**A**) Replacement strategies for *bcltf1* (deletion and complementation). (**B**) Diagnostic PCR of homokaryotic Δ*bcltf1* mutants. (**C**) Southern blot analysis of Δ*bcltf1* mutants. (**D**) Diagnostic PCR of complementation mutants. (**E**) Targeted integration of P*oliC*::*bcltf1-gfp* constructs by replacement of *bcniiA*. (**F**) Detection of *bcltf1* expression levels in the different mutants. Indicated strains were grown for 2 d in DD on solid CM. One sample per strain was exposed for 1 h to white light (+LP). For more details, see Materials and Methods.
(PDF)

Figure S2 BcLTF1-GFP fusion proteins are functional. (**A**) Expression of BcLTF1-GFP in the Δ*bcltf1* background restores light tolerance. Strains were incubated on solid CM in LL (white), LD (gray), and DD (black). Mean values and standard deviations were calculated from five colonies per strain and condition. Asterisks indicate significant differences compared to WT:B05.10 in each condition (p<0.001). (**B**) Decreased and increased expression levels of *bcltf1* impair growth under alkaline pH conditions. Strains were cultivated in LD on solid CM (pH 8) supplemented with bromothymol blue; yellow coloration indicates pH<6. (**C**) BcLTF1-GFP expressed from the native promoter in the Δ*bcltf1* background restores sclerotial development. Strains were cultivated on solid CM. (**D**) Virulence is restored by expression of BcLTF1-GFP. *P. vulgaris* plants were inoculated with non-sporulating mycelia and incubated in LD.
(PDF)

Figure S3 BcLTF1 is needed to cope with blue light. (**A**) Growth defect of Δ*bcltf1* mutants is restricted to treatment with white and blue light. Wild type and *bcltf1* mutants were incubated on solid CM under the indicated light conditions (LL) or in DD. Mean values and standard deviations were calculated from three colonies. Asterisks indicate significant differences compared to WT:B05.10 in each condition (p<0.001). (**B**) Transmission spectra of the used filters (Rosculux; http://www.rosco.com/filters/roscolux.cfm).
(PDF)

Figure S4 Conidial germination is not influenced by light. (**A**) Germination rates are not affected by light and mutations of *bcltf1*. Germination was induced by nutrients (on the left) or a hydrophobic surface (on the right). Incubation took place in DD (dark bars) or LL (bright bars). Experiments were done in triplicates. (**B**) Conidial germ tubes overexpressing BcLTF1 exhibit an altered branching pattern. Strains carrying the construct OE::*bcltf1-gfp* in a B05.10-background are heterokaryotic and form conidia with different BcLTF1 expression levels. Germ tubes that exhibit bright nuclear GFP signals are malformed, while conidia without detectable GFP fluorescence produce wild-type-like germ tubes (indicated by an asterisk). Scale bars, 25 µm.
(PDF)

Figure S5 Δ*bcltf1* mutants are impaired in invasive growth on *P. vulgaris*. (**A**) Virulence defect of Δ*bclft1* mutants does not depend on the inoculation method. Plants were inoculated with plugs of non-sporulating mycelia and incubated in DD. Black bars, 1 cm; white bars, 2 cm. (**B**) Virulence of wild type and *bcltf1* mutants is not affected by the illumination condition. Plants were inoculated with conidial suspensions or non-sporulating mycelia and incubated in DD or LD. Mean values and standard deviations were calculated from twelve lesions per strain and condition. Asterisks indicate significant differences compared to WT:B05.10 in each condition (p<0.001). (**C**) Trypan blue staining indicates decreased proliferation of Δ*bcltf1* mutants during infection. Primary leaves were inoculated with conidial suspensions, detached and stained at 48 and 72 hpi. Black bars, 3 mm; white bars, 200 µm.
(PDF)

Figure S6 Ascorbic acid improves growth of Δ*bcltf1* mutants on minimal medium. Strains were incubated for three days in LD and DD on solid CD containing 3 g/l NaNO_3_ or 1 g/l (NH_4_)_2_SO_4_ as nitrogen sources, with and without ascorbic acid (AA). Mean values and standard deviations were calculated from four colonies per strain and condition. Asterisks indicate significant differences compared to WT:B05.10 in each condition (p<0.001).
(PDF)

Figure S7 Absence of BcLTF1 affects the expression of secondary metabolism-related genes. Relative gene expression depicted by color scale; shades of green and red indicate under- and overexpression, respectively. Asterisks indicate differentially expressed genes (>2-fold, p<0.05) in WT-D/+LP (orange) or WT/Δ*bcltf1* (black). (**A**) Seventeen out of the 38 expressed key enzyme-encoding genes are differentially expressed in the Δ*bcltf1* mutants. (**B**) Genes required for biosynthesis of BOT and BOA are overexpressed in the absence of *bcltf1*.
(PDF)

Figure S8 Melanin biosynthesis is up-regulated in Δ*bcltf1* mutants. (**A**) Proposed pathway for biosynthesis of DHN-melanin in *B. cinerea*. Treatment with tricyclazole results in the accumulation the shunt product flaviolin. (**B**) Genes encoding melanogenic enzymes are distributed in genome. *B. cinerea* possesses two highly similar PKS-encoding genes (*bcpks12, bcpks13*); the first one is physically linked with *bcmtf1* encoding a Zn2Cys6-TF. (**C**) Expression levels of melanogenic genes are diversely affected by the Δ*bcltf1* mutation. Asterisks indicate genes with fold change >1.5, p<0.05 in WT/Δ*bcltf1*. (**D**) *Bcltf1* deletion mutants produce melanin in excess. Strains were incubated in liquid CD+0.2% yeast extract for 3 d in DD. (**E**) *Bcpks12* and *bcpks13* are differentially expressed during the lifecycle. Strains were cultivated on solid CM in DD (gray) or LD (yellow) and harvested at 2, 4 or 6 dpi. Sclerotia (S) were collected at 10 dpi. *Bcpks12* was exclusively expressed in sclerotia (*).
(PDF)

Figure S9 Biosynthesis of carotenoids is induced by light. (**A**) Proposed pathway for carotenoid biosynthesis in *B. cinerea*. Genetic makeup may allow for the production of neurosporaxanthin and retinal as end products of a branched pathway. (**B**) The three genes required for the retinal biosynthesis are physically linked with the opsin-encoding *bop2*. Gene clusters of *B. cinerea* and *F. fujikuroi* are organized in the same way. (**C**) Expression levels of the retinal cluster genes increase upon illumination. Asterisks indicate genes with fold change >2, p<0.05 in WT:B05.10-D/+LP. Expression levels in response to light are much lower in the Δ*bcltf1* background suggesting decreased carotenoid production in the deletion mutants.
(PDF)

Figure S10 Deletion of *bcltf1* leads to changes in the mitochondria – indications for alternative respiration. *B. cinerea* genes encoding enzyme activities indicated in red are overexpressed (>1.5x, p<0.05) in the Δ*bcltf1* mutants irrespective of the illumination condition (for more details see Table S4). Abbreviations: I – NADH dehydrogenase complex, II – succinate dehydrogenase complex, Q – ubiquinone, III – cytochrome bc_1_ complex, cyt c – cytochrome c, IV – cytochrome c oxidase complex (COX), V – ATP synthase, AOX – alternative oxidase, CCP – cytochrome c peroxidase, NDE – alternative NADH dehydrogenase, external, NDI – alternative NADH dehydrogenase, internal, UCP – uncoupling protein, COX17 – COX copper chaperone. The gray arrow indicates the electron flux during cytochrome respiration (ETC) accompanied by O_2_^−^ formation at complexes I and III; the red arrows indicate the electron flux during alternative respiration, complexes I and III are bypassed by NDE/NDI and AOX, respectively.
(PDF)

Table S1 Oligonucleotides used for sequencing and mutant construction in this study.
(PDF)

Table S2 List of differentially expressed genes in response to light and/or due to Δ*bcltf1*.
(XLSX)

Table S3 Genes subjected to hierarchical clustering analyses.
(XLSX)

Table S4 Expression patterns of genes whose products may affect the cellular ROS homoeostasis in *B. cinerea*.
(XLSX)
