# Supplementary figures and images for: The Transcription Factor BcLTF1 Regulates Virulence and Light Responses in the Necrotrophic Plant Pathogen Botrytis cinerea
Source: PLoS Genet. 2014 Jan 9;10(1):e1004040. doi: 10.1371/journal.pgen.1004040 (PMC3886904; doi:10.1371/journal.pgen.1004040)

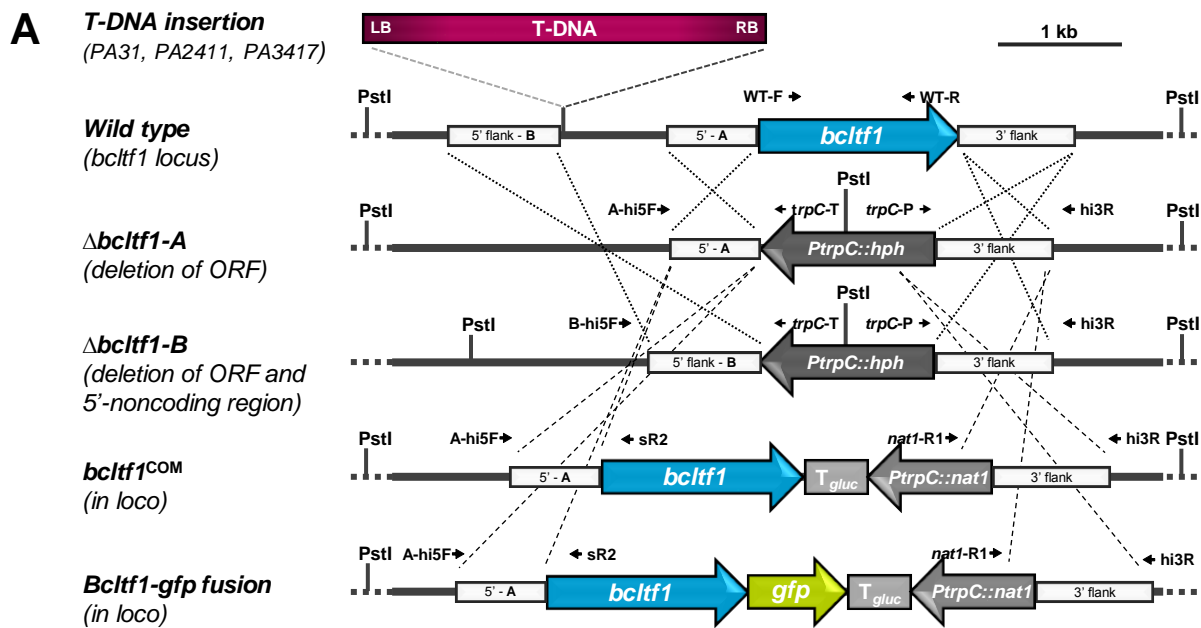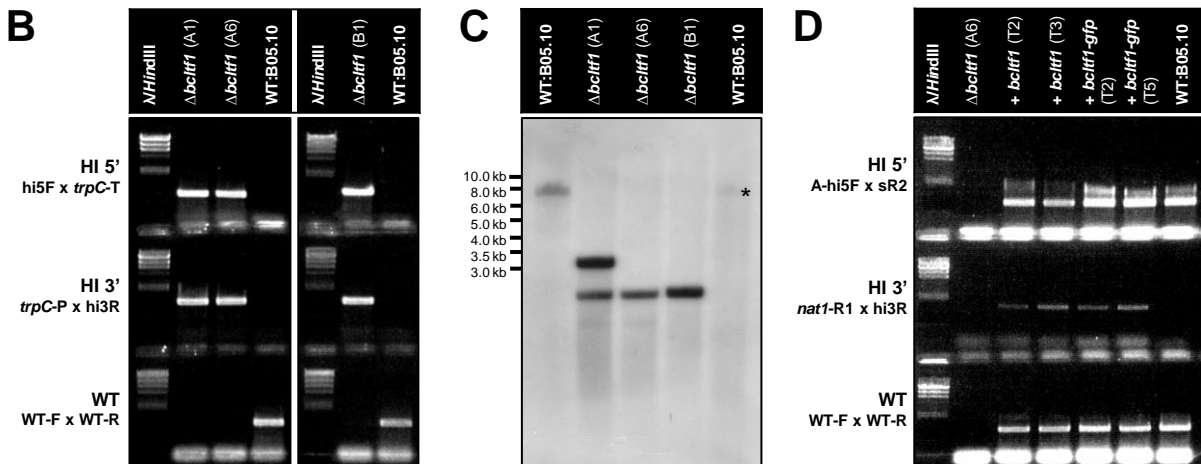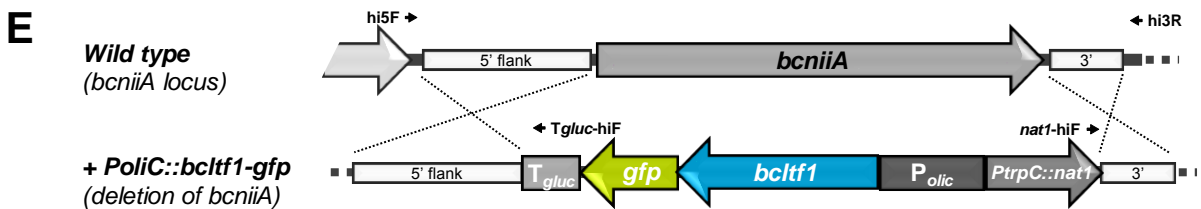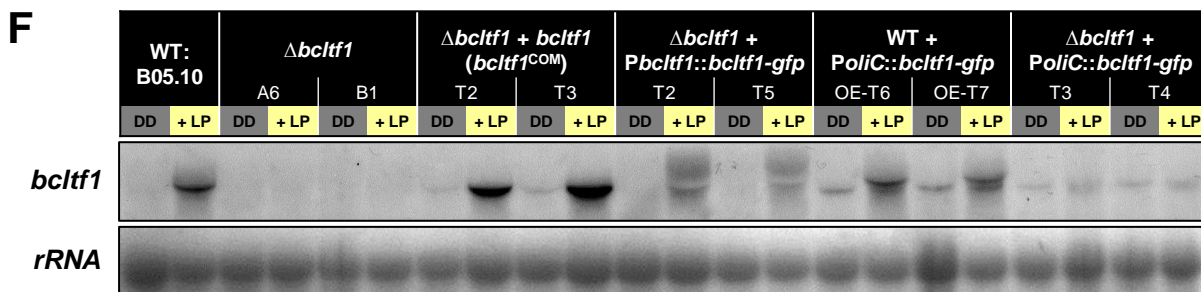

Supplement: Figure S1 — Construction of bcltf1 mutants. (A) Replacement strategies for bcltf1 (deletion and complementation). (B) Diagnostic PCR of homokaryotic Δbcltf1 mutants. (C) Southern blot analysis of Δbcltf1 mutants. (D) Diagnostic PCR of complementation mutants. (E) Targeted integration of PoliC::bcltf1-gfp constructs by replacement of bcniiA. (F) Detection of bcltf1 expression levels in the different mutants. Indicated strains were grown for 2 d in DD on solid CM. One sample per strain was exposed for 1 h to white light (+LP). For more details, see Materials and Methods. (PDF) [file pgen.1004040.s001.pdf]

**A**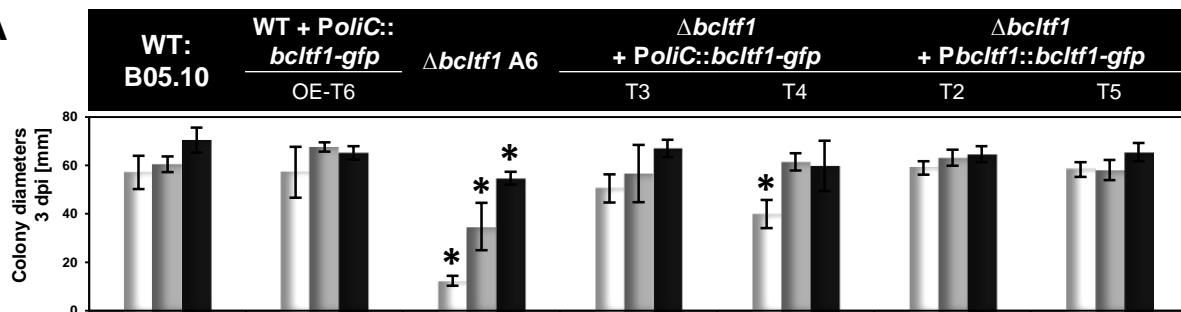**B**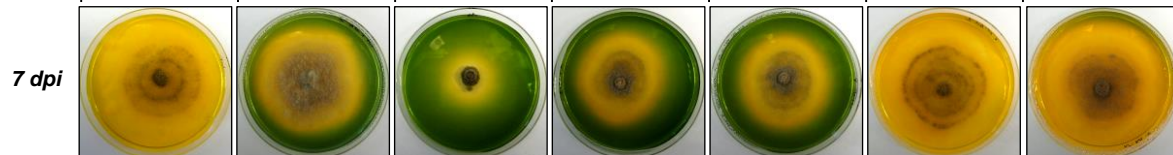**C**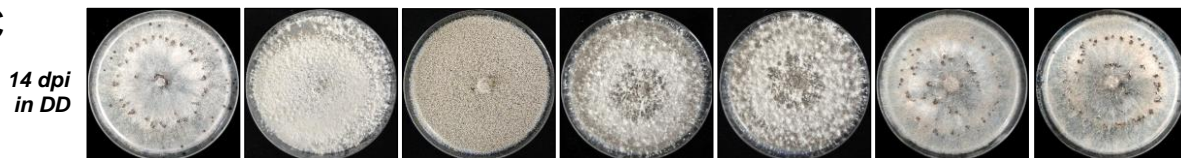**D**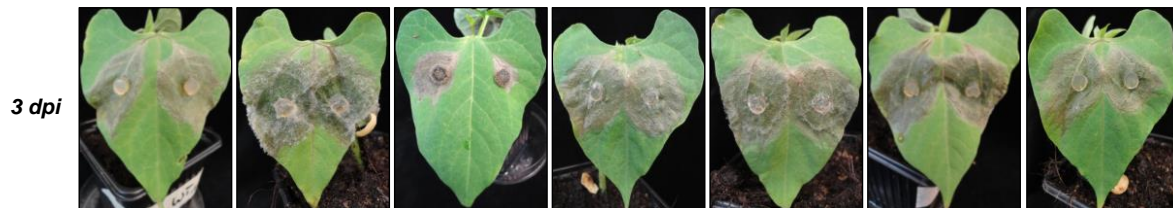

Supplement: Figure S2 — BcLTF1-GFP fusion proteins are functional. (A) Expression of BcLTF1-GFP in the Δbcltf1 background restores light tolerance. Strains were incubated on solid CM in LL (white), LD (gray), and DD (black). Mean values and standard deviations were calculated from five colonies per strain and condition. Asterisks indicate significant differences compared to WT:B05.10 in each condition (p<0.001). (B) Decreased and increased expression levels of bcltf1 impair growth under alkaline pH conditions. Strains were cultivated in LD on solid CM (pH 8) supplemented with bromothymol blue; yellow coloration indicates pH<6. (C) BcLTF1-GFP expressed from the native promoter in the Δbcltf1 background restores sclerotial development. Strains were cultivated on solid CM. (D) Virulence is restored by expression of BcLTF1-GFP. P. vulgaris plants were inoculated with non-sporulating mycelia and incubated in LD. (PDF) [file pgen.1004040.s002.pdf]

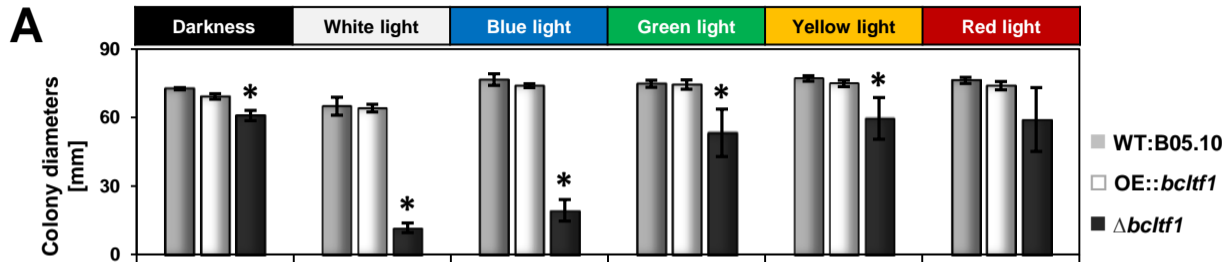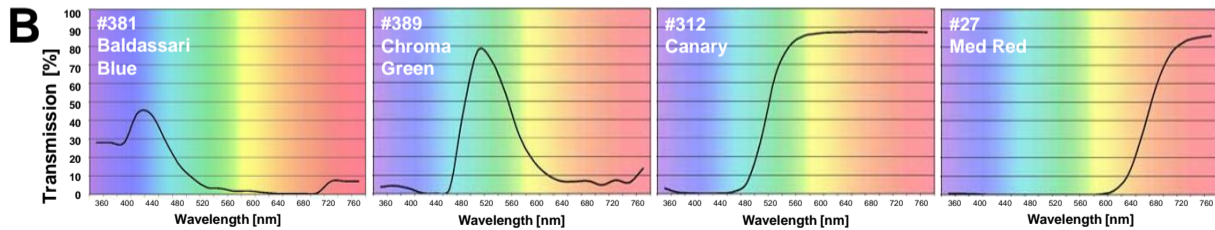

Supplement: Figure S3 — BcLTF1 is needed to cope with blue light. (A) Growth defect of Δbcltf1 mutants is restricted to treatment with white and blue light. Wild type and bcltf1 mutants were incubated on solid CM under the indicated light conditions (LL) or in DD. Mean values and standard deviations were calculated from three colonies. Asterisks indicate significant differences compared to WT:B05.10 in each condition (p<0.001). (B) Transmission spectra of the used filters (Rosculux; http://www.rosco.com/filters/roscolux.cfm). (PDF) [file pgen.1004040.s003.pdf]

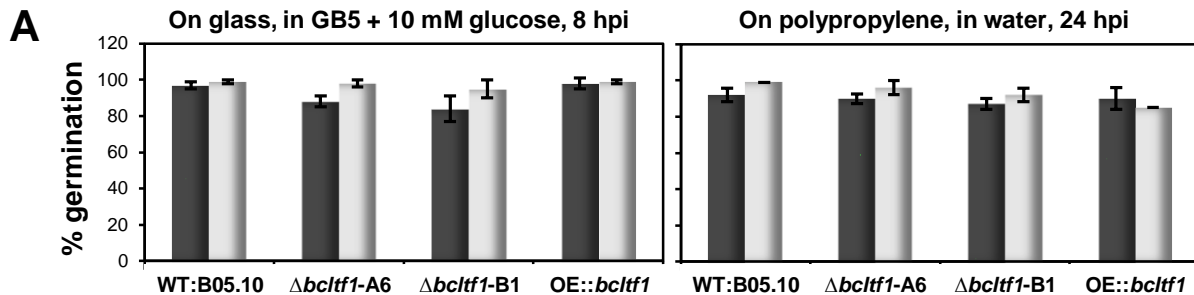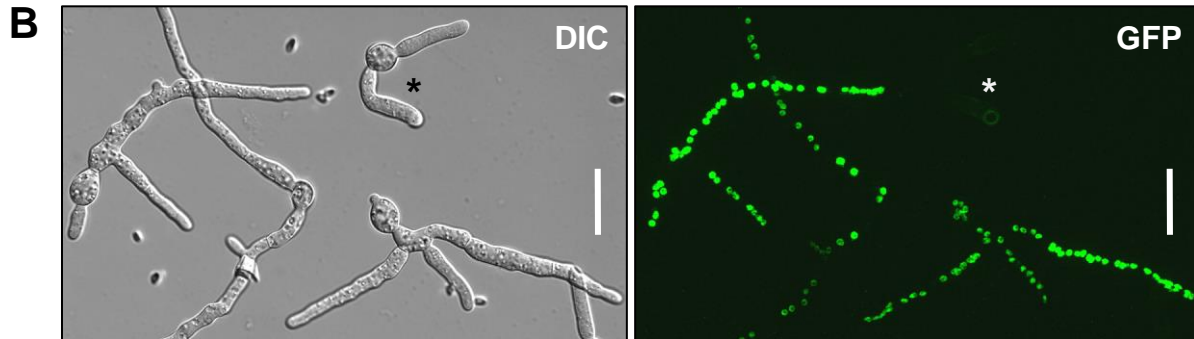

Supplement: Figure S4 — Conidial germination is not influenced by light. (A) Germination rates are not affected by light and mutations of bcltf1. Germination was induced by nutrients (on the left) or a hydrophobic surface (on the right). Incubation took place in DD (dark bars) or LL (bright bars). Experiments were done in triplicates. (B) Conidial germ tubes overexpressing BcLTF1 exhibit an altered branching pattern. Strains carrying the construct OE::bcltf1-gfp in a B05.10-background are heterokaryotic and form conidia with different BcLTF1 expression levels. Germ tubes that exhibit bright nuclear GFP signals are malformed, while conidia without detectable GFP fluorescence produce wild-type-like germ tubes (indicated by an asterisk). Scale bars, 25 µm. (PDF) [file pgen.1004040.s004.pdf]

**A**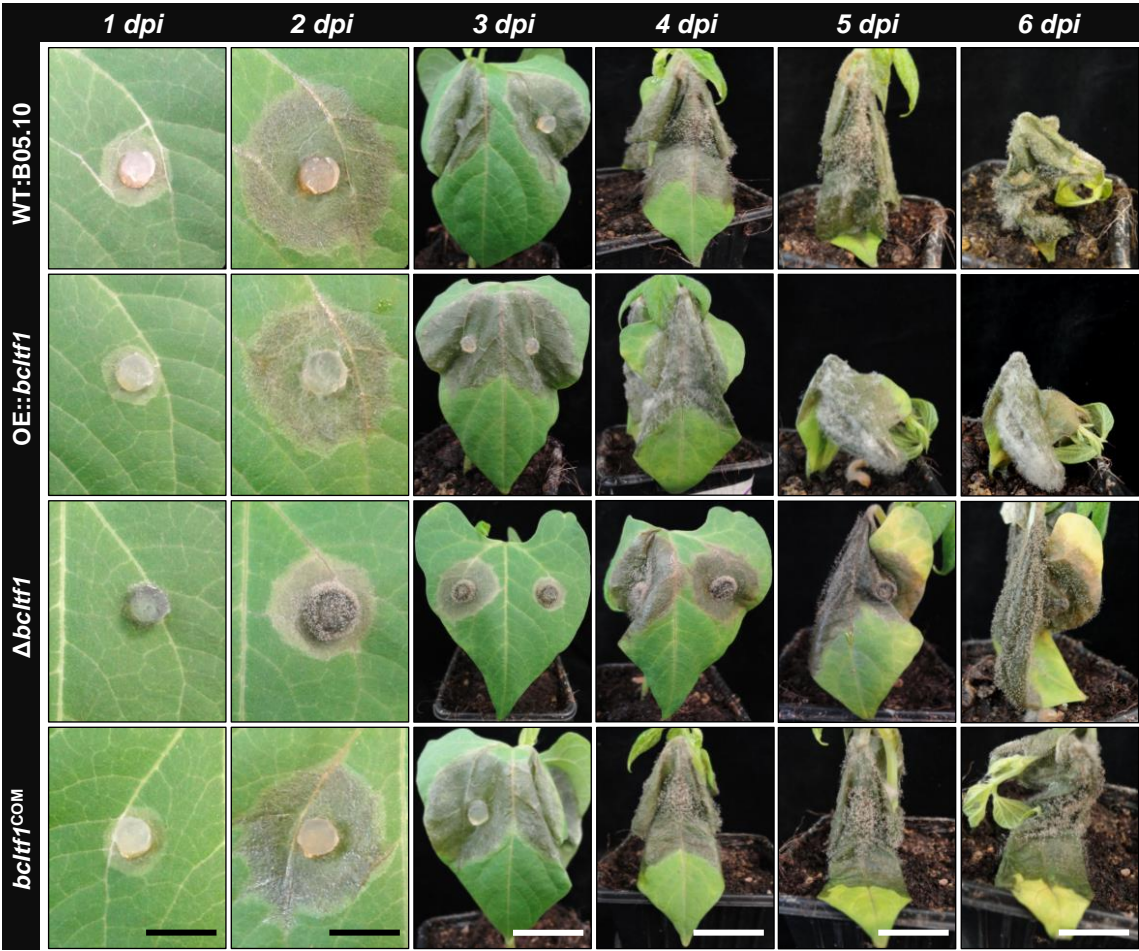**B**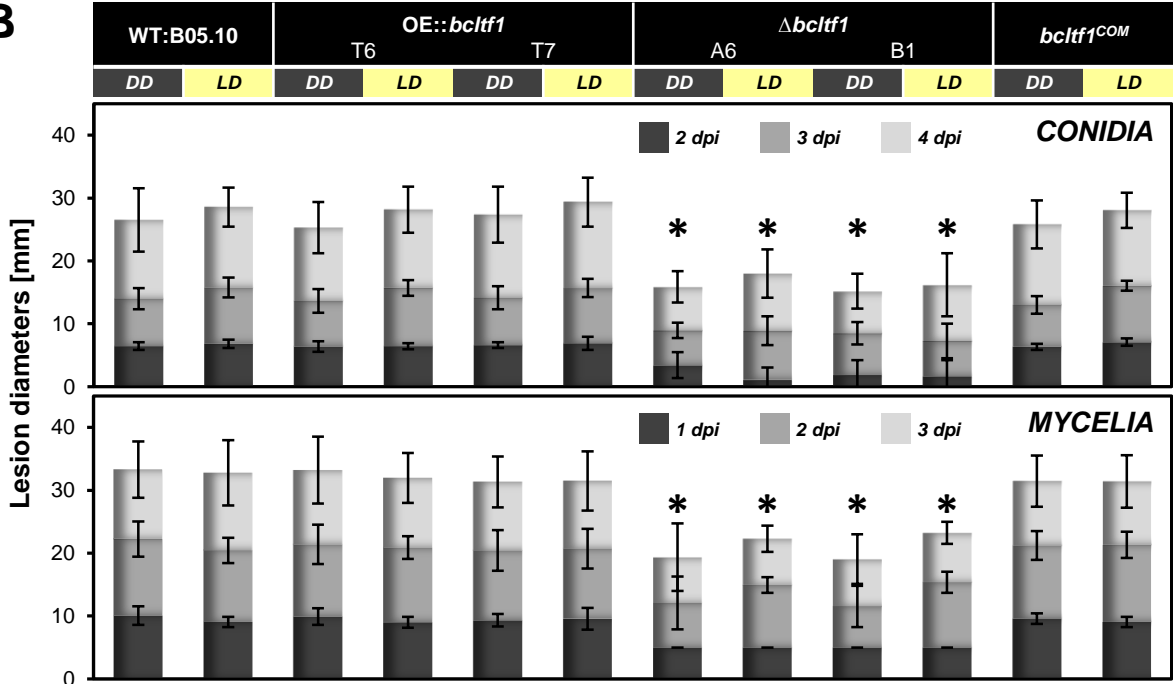**C**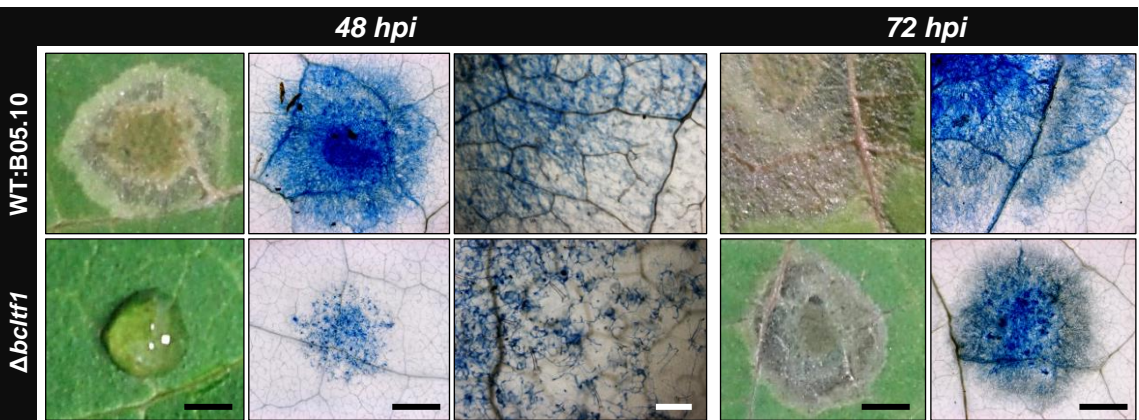

Supplement: Figure S5 — Δbcltf1 mutants are impaired in invasive growth on P. vulgaris. (A) Virulence defect of Δbclft1 mutants does not depend on the inoculation method. Plants were inoculated with plugs of non-sporulating mycelia and incubated in DD. Black bars, 1 cm; white bars, 2 cm. (B) Virulence of wild type and bcltf1 mutants is not affected by the illumination condition. Plants were inoculated with conidial suspensions or non-sporulating mycelia and incubated in DD or LD. Mean values and standard deviations were calculated from twelve lesions per strain and condition. Asterisks indicate significant differences compared to WT:B05.10 in each condition (p<0.001). (C) Trypan blue staining indicates decreased proliferation of Δbcltf1 mutants during infection. Primary leaves were inoculated with conidial suspensions, detached and stained at 48 and 72 hpi. Black bars, 3 mm; white bars, 200 µm. (PDF) [file pgen.1004040.s005.pdf]

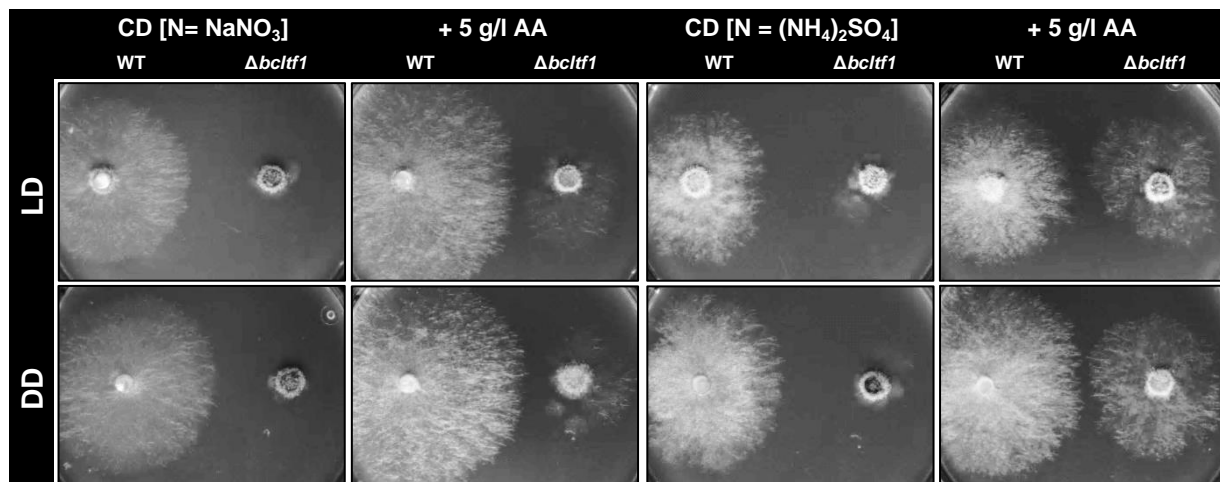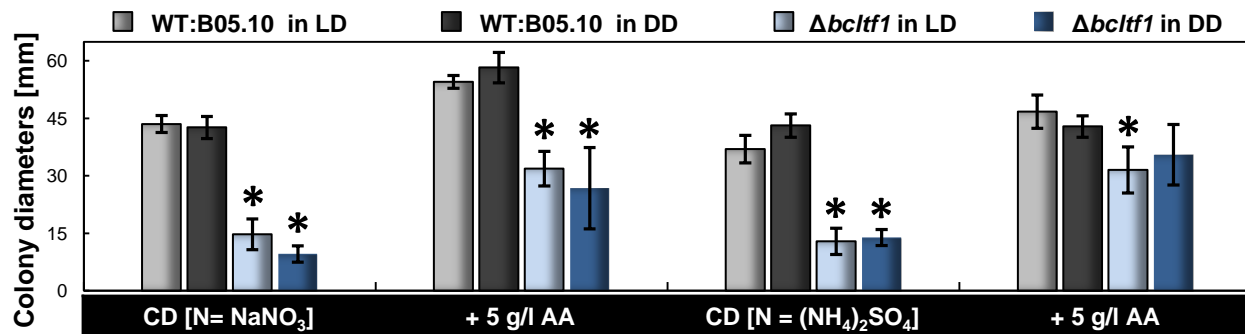

Supplement: Figure S6 — Ascorbic acid improves growth of Δbcltf1 mutants on minimal medium. Strains were incubated for three days in LD and DD on solid CD containing 3 g/l NaNO3 or 1 g/l (NH4)2SO4 as nitrogen sources, with and without ascorbic acid (AA). Mean values and standard deviations were calculated from four colonies per strain and condition. Asterisks indicate significant differences compared to WT:B05.10 in each condition (p<0.001). (PDF) [file pgen.1004040.s006.pdf]

**A**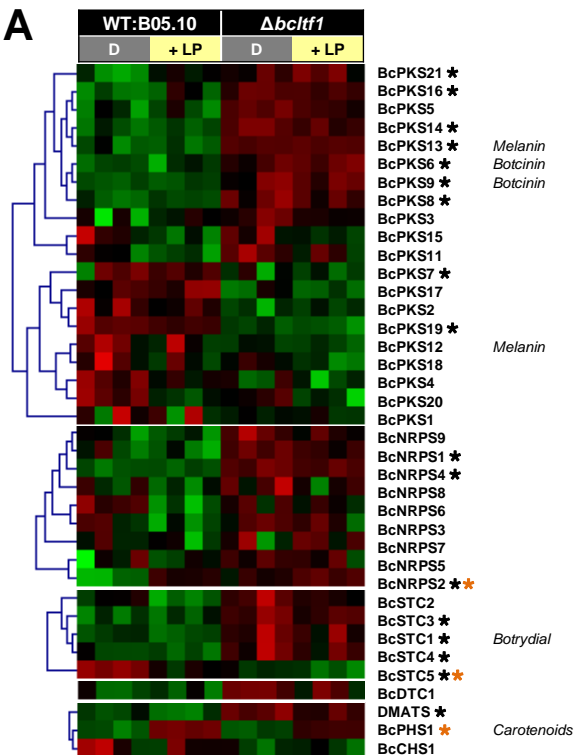**B**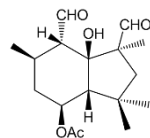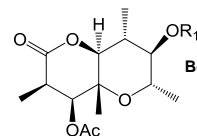

### Botrydial (BOT) gene cluster

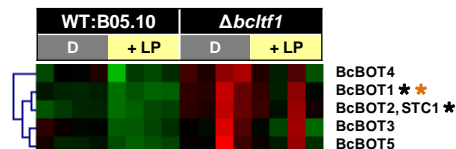

### Botcinic acid (BOA) gene cluster

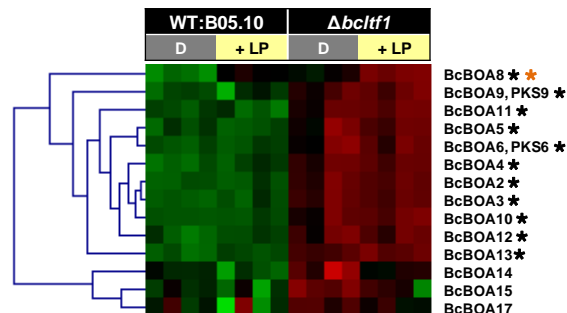

Supplement: Figure S7 — Absence of BcLTF1 affects the expression of secondary metabolism-related genes. Relative gene expression depicted by color scale; shades of green and red indicate under- and overexpression, respectively. Asterisks indicate differentially expressed genes (>2-fold, p<0.05) in WT-D/+LP (orange) or WT/Δbcltf1 (black). (A) Seventeen out of the 38 expressed key enzyme-encoding genes are differentially expressed in the Δbcltf1 mutants. (B) Genes required for biosynthesis of BOT and BOA are overexpressed in the absence of bcltf1. (PDF) [file pgen.1004040.s007.pdf]

**A**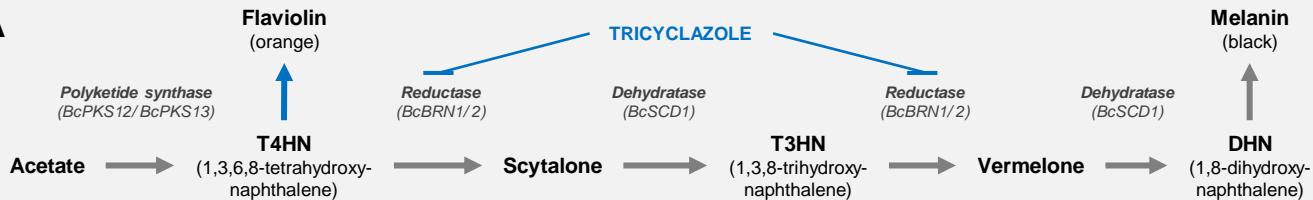**B**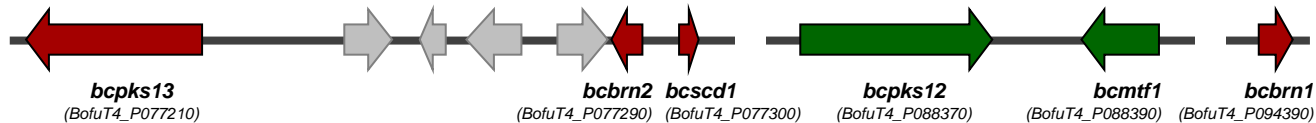**C**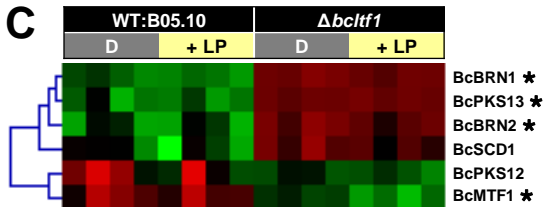**D**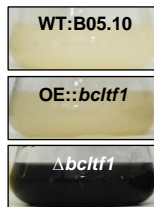**E**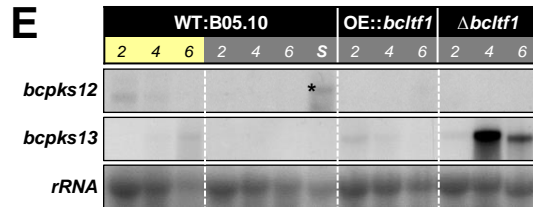

Supplement: Figure S8 — Melanin biosynthesis is up-regulated in Δbcltf1 mutants. (A) Proposed pathway for biosynthesis of DHN-melanin in B. cinerea. Treatment with tricyclazole results in the accumulation the shunt product flaviolin. (B) Genes encoding melanogenic enzymes are distributed in genome. B. cinerea possesses two highly similar PKS-encoding genes (bcpks12, bcpks13); the first one is physically linked with bcmtf1 encoding a Zn2Cys6-TF. (C) Expression levels of melanogenic genes are diversely affected by the Δbcltf1 mutation. Asterisks indicate genes with fold change >1.5, p<0.05 in WT/Δbcltf1. (D) Bcltf1 deletion mutants produce melanin in excess. Strains were incubated in liquid CD+0.2% yeast extract for 3 d in DD. (E) Bcpks12 and bcpks13 are differentially expressed during the lifecycle. Strains were cultivated on solid CM in DD (gray) or LD (yellow) and harvested at 2, 4 or 6 dpi. Sclerotia (S) were collected at 10 dpi. Bcpks12 was exclusively expressed in sclerotia (*). (PDF) [file pgen.1004040.s008.pdf]

**A**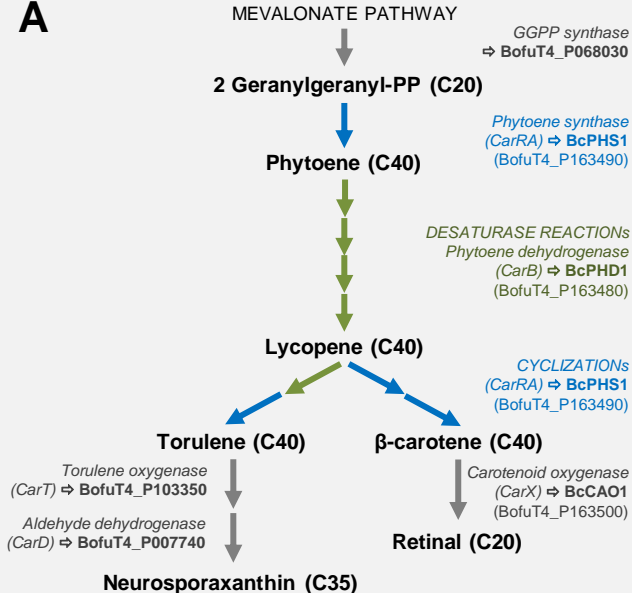**B**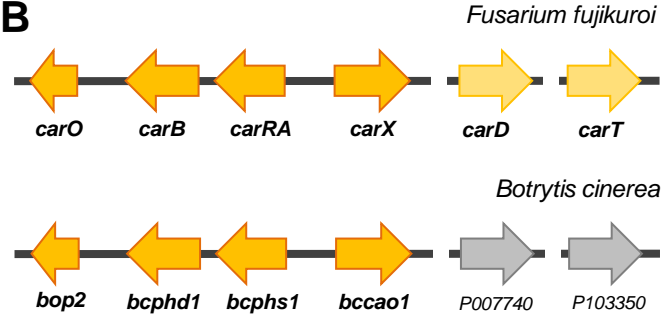**C**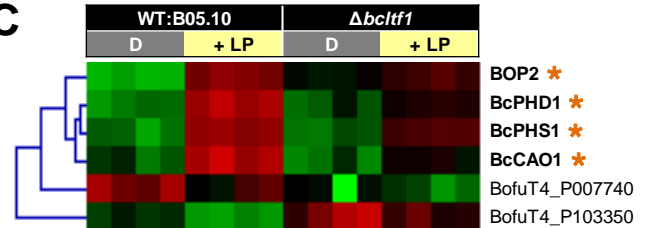

Supplement: Figure S9 — Biosynthesis of carotenoids is induced by light. (A) Proposed pathway for carotenoid biosynthesis in B. cinerea. Genetic makeup may allow for the production of neurosporaxanthin and retinal as end products of a branched pathway. (B) The three genes required for the retinal biosynthesis are physically linked with the opsin-encoding bop2. Gene clusters of B. cinerea and F. fujikuroi are organized in the same way. (C) Expression levels of the retinal cluster genes increase upon illumination. Asterisks indicate genes with fold change >2, p<0.05 in WT:B05.10-D/+LP. Expression levels in response to light are much lower in the Δbcltf1 background suggesting decreased carotenoid production in the deletion mutants. (PDF) [file pgen.1004040.s009.pdf]

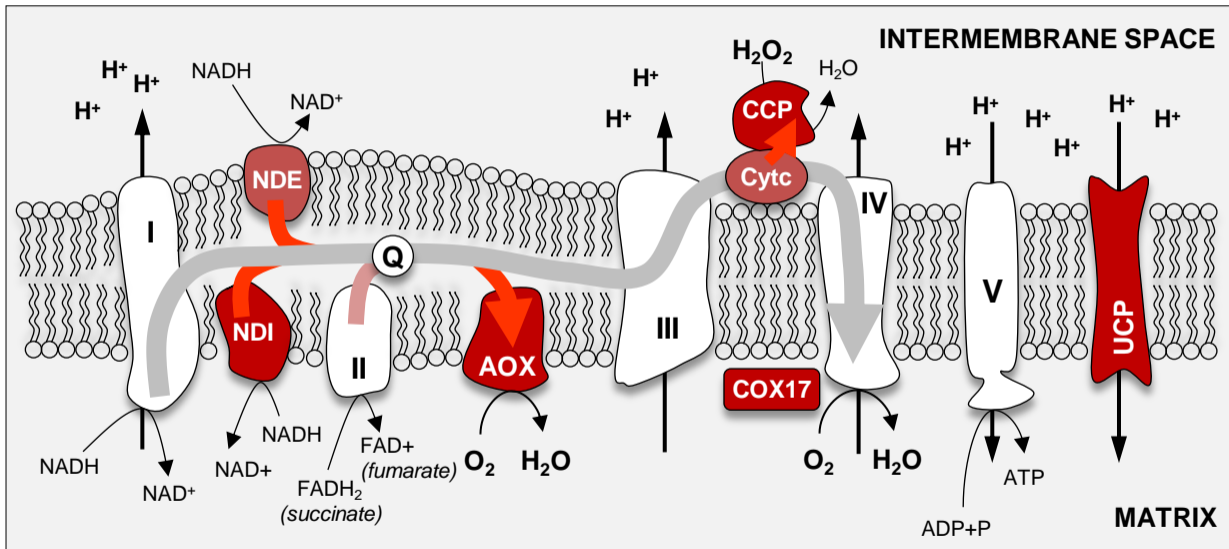

Supplement: Figure S10 — Deletion of bcltf1 leads to changes in the mitochondria – indications for alternative respiration. B. cinerea genes encoding enzyme activities indicated in red are overexpressed (>1.5x, p<0.05) in the Δbcltf1 mutants irrespective of the illumination condition (for more details see Table S4). Abbreviations: I – NADH dehydrogenase complex, II – succinate dehydrogenase complex, Q – ubiquinone, III – cytochrome bc1 complex, cyt c – cytochrome c, IV – cytochrome c oxidase complex (COX), V – ATP synthase, AOX – alternative oxidase, CCP – cytochrome c peroxidase, NDE – alternative NADH dehydrogenase, external, NDI – alternative NADH dehydrogenase, internal, UCP – uncoupling protein, COX17 – COX copper chaperone. The gray arrow indicates the electron flux during cytochrome respiration (ETC) accompanied by O2 − formation at complexes I and III; the red arrows indicate the electron flux during alternative respiration, complexes I and III are bypassed by NDE/NDI and AOX, respectively. (PDF) [file pgen.1004040.s010.pdf]
