# Supplementary material for: The Transcription Factor BcLTF1 Regulates Virulence and Light Responses in the Necrotrophic Plant Pathogen Botrytis cinerea
Source: PLoS Genet. 2014 Jan 9;10(1):e1004040. doi: 10.1371/journal.pgen.1004040 (PMC3886904; doi:10.1371/journal.pgen.1004040)
Supplement: Table S1 — Oligonucleotides used for sequencing and mutant construction in this study. (PDF) [file pgen.1004040.s011.pdf]

Primer	Sequence (5’ → 3’)	Features	Used for
***Bcltf1*-F1**	GTACTGTGAGATTCTAAAGCTCG	*Bcltf1 locus*	Sequencing – 2,969 bp upstream of *bcltf1*
***Bcltf1*-F2**	GTGAGTGTGCTTGGTCTCAATTAG	*Bcltf1 locus*	Sequencing – 2,240 bp upstream of *bcltf1*
***Bcltf1*-F3**	GCCATGTCGTTCGTCCACTTTG	*Bcltf1 locus*	Sequencing – 1,856 bp upstream of *bcltf1*
***Bcltf1*-F4**	GCCATGACATTATCCATCTCTTAG	*Bcltf1 locus*	Sequencing – 1,473 bp upstream of *bcltf1*
***Bcltf1*-F5**	CGATGCTGCCGCTTCCTCCC	*Bcltf1 locus*	Sequencing – 1,269 bp upstream of *bcltf1*
***Bcltf1*-F6**	GCTCACCTGCCCTGTTGTGTACC	*Bcltf1 locus*	Sequencing – 0,991 bp upstream of *bcltf1*
***Bcltf1*-F7**	GTGTAATTCAGCCTCATTCCGATCG	*Bcltf1 locus*	Sequencing – 0,577 bp upstream of *bcltf1*
***Bcltf1*-F8**	GTCTTTCAGCCGCACACCAATTAG	*Bcltf1 locus*	Sequencing – 0,248 bp upstream of *bcltf1*
***Bcltf1*-A-5F**	gtaacgccagggttttcccagtcacgacg-CACAGAGTCAATTGGGTTCATT	pRS426-5F - *bcltf1*	Cloning – amplification of 5' flank for KO construct A
***Bcltf1*-A-5R**	atccacttaacgttactgaaatctccaac-CTCGAATCTGAACAGATTAGATG	*Hph - bcltf1*	Cloning – amplification of 5' flank for KO construct A
***Bcltf1*-3F**	ctccttcaatatcatcttctgtctccgac-GCCTTTATCAGCAGTACCTAC	P*trpC - bcltf1*	Cloning – amplification of 3' flank for KO constructs
***Bcltf1*-3R**	gcggataacaatttcacacaggaaacagc-CATCTGCGATCCGTGGACCC	pRS426-3R - *bcltf1*	Cloning – amplification of 3' flank for KO constructs
***Bcltf1*-B-5F**	gtaacgccagggttttcccagtcacgacg-GACCTCGCTCGCCTACATCAG	pRS426-5F - *bcltf1*	Cloning – amplification of 5' flank for KO construct B
***Bcltf1*-B-5R**	atccacttaacgttactgaaatctccaac-GGTATGGATCATATCGATGGTC	*Hph - bcltf1*	Cloning – amplification of 5' flank for KO construct B
***Hph*-F**	GTCGGAGACAGAAGATGATATTGAAGGAGC	P*trpC*	Cloning – amplification of P*trpC*::*hph* and P*trpC*::*nat1*
***Hph*-R**	GTTGGAGATTTCAGTAACGTTAAGTGGAT	*Hph*	Cloning – amplification of P*trpC*::*hph*
***Bcltf1*-A-hi5F**	CTCACGAAACGCACCCTACGATTC	*Bcltf1 locus*	Diagnostic PCR – homologous integration at 5' (KO-A)
***Bcltf1*-B-hi5F**	GATCGCGAACAGTTAAGGTATGG	*Bcltf1 locus*	Diagnostic PCR – homologous integration at 5' (KO-B)
***TrpC*-P**	CCTCCACTAGCTCCAGCCAAGCCC	P*trpC*::*hph*	Diagnostic PCR – homologous integration at 5' (KO-A/B)
***Bcltf1*-hi3R**	CAGCGCACAGCGCATAGTGCATAC	*Bcltf1 locus*	Diagnostic PCR – homologous integration at 3' (KO-A/B)
***TrpC*-T**	GGAATAGAGTAGATGCCGACCGG	P*trpC*::*hph*	Diagnostic PCR – homologous integration at 3' (KO-A/B)
***Bcltf1*-WT-F**	GGCTGCACCACCGACTGCCAGTCCTG	*Bcltf1 ORF*	Diagnostic PCR – detection of *bcltf1* alleles
***Bcltf1*-WT-R**	CGTCGGCTGCATGCTGCTGCTGTAC	*Bcltf1 ORF*	Diagnostic PCR – detection of *bcltf1* alleles
***Bcltf1*-PoliC-F**	ctccatcacatcacaatcgatccaacc-ATGGAGGGTGCGGAAAGTGGC	PoliC - *bcltf1*	Cloning – amplification of *bcltf1*
***Bcltf1*-GFP-R**	cttacctcacccttggaaaccat-TGACCGTGGTGAATGATCCTC	*Gfp - bcltf1*	Cloning – amplification of *bcltf1*
***BcniiA*-hi5F**	GCGGGGTATGGCAGCATGAGTG	*BcniiA locus*	Diagnostic PCR – homologous integration at 5'
**T*gluc*-hiF**	CATACGTACATCTGATTTGACAACC	T*gluc*	Diagnostic PCR – homologous integration at 5'
***BcniiA*-hi3R**	CTTATAGCAAGCGCGATGTGTATC	*BcniiA locus*	Diagnostic PCR – homologous integration at 3'
***Nat1*-hiF**	CGGCGAGCAGGCGCTCTACATGAGC	*Nat1*	Diagnostic PCR – homologous integration at 3'
***BcniiA*-WT-F**	GGTTGAGGTGGTGGAAGATTTG	*BcniiA ORF*	Diagnostic PCR – detection of *bcniiA* alleles
***BcniiA*-WT-R**	CGACCACCAAGCCTCCAGCATC	*BcniiA ORF*	Diagnostic PCR – detection of *bcniiA* alleles
***Bcltf1*-Tgluc-R**	catacatcttatctacatacg-CTATGACCGTGGTGAATGATCC	T*gluc - bcltf1*	Cloning – amplification of *bcltf1*
***Gfp*-F**	ATGGTTTCCAAGGGTGAGg	*Gfp*	Cloning – amplification of *gfp*
**T*gluc*-F2**	gcggccgctTAGCGTATGTAGATAAGATGTATG	T*gluc*	Cloning – amplification of T*gluc*
**T*gluc*-nat1-R**	ccacttaacgttactgaaatctccaac-ATCTTGTTGGGGGGAAGGGGT	T*gluc - nat1*	Cloning – amplification of P*trpC*::*nat1*
***Hph*R-T2**	gttggagatttcagtaacgttaagtggat-CGTATCTTATCGAGATCCTGAACACC	*Hph - nat1*	Cloning – amplification of P*trpC*::*nat1*
***Nat1*-R1**	CAGTGCCTCGATGGCCTCGGCGTC	*Nat1*	Diagnostic PCR – homologous integration at 3'
***Bcltf1*-sR2**	CACGGTTCATGATGGAGAGGATGC	*Bcltf1 locus*	Diagnostic PCR – homologous integration 5'
